# ELK4 Promotes Colorectal Cancer Progression by Activating the Neoangiogenic Factor LRG1 in a Noncanonical SP1/3‐Dependent Manner

**DOI:** 10.1002/advs.202303378

**Published:** 2023-10-02

**Authors:** Zhehui Zhu, Yuegui Guo, Yun Liu, Rui Ding, Zhenyu Huang, Wei Yu, Long Cui, Peng Du, Ajay Goel, Chen‐Ying Liu

**Affiliations:** ^1^ Department of Colorectal and Anal Surgery Shanghai Colorectal Cancer Research Center Xinhua Hospital Shanghai Jiao Tong University School of Medicine Shanghai 200092 China; ^2^ Department of General Surgery State Key Laboratory of Genetic Engineering School of Life Sciences Zhongshan Hospital Fudan University Shanghai 200438 China; ^3^ Center for Gastrointestinal Research Baylor Scott & White Research Institute and Charles A. Sammons Cancer Center Baylor University Medical Center Department of Molecular Diagnostics and Experimental Therapeutics Beckman Research Institute of City of Hope Comprehensive Cancer Center Duarte CA 91010 USA

**Keywords:** colorectal cancer, ELK4, LRG1, SP1, SP3

## Abstract

Although the MAPK/MEK/ERK pathway is prevalently activated in colorectal cancer (CRC), MEK/ERK inhibitors show limited efficiency in clinic. As a downstream target of MAPK, ELK4 is thought to work primarily by forming a complex with SRF. Whether ELK4 can serve as a potential therapeutic target is unclear and the transcriptional regulatory mechanism has not been systemically analyzed. Here, it is shown that ELK4 promotes CRC tumorigenesis. Integrated genomics‐ and proteomics‐based approaches identified SP1 and SP3, instead of SRF, as cooperative functional partners of ELK4 at genome‐wide level in CRC. Serum‐induced phosphorylation of ELK4 by MAPKs facilitated its interaction with SP1/SP3. The pathological neoangiogenic factor LRG1 is identified as a direct target of the ELK4‐SP1/SP3 complex. Furthermore, targeting the ELK4‐SP1/SP3 complex by combination treatment with MEK/ERK inhibitor and the relatively specific SP1 inhibitor mithramycin A (MMA) elicited a synergistic antitumor effect on CRC. Clinically, ELK4 is a marker of poor prognosis in CRC. A 9‐gene prognostic model based on the ELK4‐SP1/3 complex‐regulated gene set showed robust prognostic accuracy. The results demonstrate that ELK4 cooperates with SP1 and SP3 to transcriptionally regulate LRG1 to promote CRC tumorigenesis in an SRF‐independent manner, identifying the ELK4‐SP1/SP3 complex as a potential target for rational combination therapy.

## Graphical abstract

This study reveals that ELK4 forms a novel complex with SP1/3, instead of SRF, to transcriptionally regulate the pro‐angiogenic LRG1 to promote CRC tumorigenesis. Targeting the ELK4‐SP1/3 transcriptional complex with combinations of MEK/ERK inhibitors and SP1 inhibitors is a promising strategy for CRC treatment. A 9‐gene prognostic model based on the ELK4‐SP1/3‐regulated gene set shows robust prognostic accuracy.

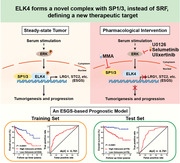

## 1 Introduction

Colorectal cancer (CRC) is the second most common cause of cancer‐related death and the third most frequently diagnosed cancer in the United States.^[^ ^1^ ^]^ Many genetic and epigenetic alterations lead to transcriptome dysregulation, which contributes to CRC tumorigenesis and progression.^[^ ^2^ ^]^ A mechanistic understanding of the dysregulated transcriptome and identification of the underlying key dysregulated transcription factors in CRC could help in the development of novel effective targeted therapeutic approaches and would greatly benefit the clinical outcomes of CRC patients.

The transcription factor ELK4 belongs to the ternary complex factor (TCF) subfamily of ETS transcription factors, which includes ELK1 and NET.^[^ ^3^ ^]^ Unlike other members of the ETS family, both ELK1 and ELK4 are thought to function primarily by complexing with another transcription factor, serum response factor (SRF), and, in response to growth factor stimulation, simultaneously activating the expression of immediate early genes (IEGs) such as egr‐1 and c‐fos.^[^ ^4^, ^5^ ^]^ The role of ELK4 in tumorigenesis is dependent on the cellular context. The chimeric fusion SLC45A4‐ELK4 transcript was identified in prostate cancer, but further study revealed that SLC45A4‐ELK4 functions as a lncRNA to promote prostate cancer cell proliferation.^[^ ^6^ ^]^ In addition, ELK4 has been reported to activate the proto‐oncogene c‐fos^[^ ^7^ ^]^ and the antiapoptotic protein Mcl‐1^[^ ^8^ ^]^ to maintain the malignant phenotype in melanoma and promote tumor formation in glioblastoma, respectively. ELK4 was also found to be a tumor suppressor gene that suppresses the proliferation and promotes the apoptosis of vestibular schwannoma cells.^[^ ^9^ ^]^ Previously, we revealed the protumorigenic role of LAMB3 in CRC and showed that transcriptional upregulation of LAMB3 is regulated by ELK4, suggesting the potential oncogenic role of ELK4 in CRC.^[^ ^10^ ^]^ However, the biological function and regulatory mechanism of ELK4 in CRC remain unknown.

Leucine‐rich‐alpha‐2‐glycoprotein 1 (LRG1) is a newly identified regulator of pathogenic angiogenesis that also plays a vital role in tumor progression in various cancers, including CRC.^[^ ^11^, ^12^ ^]^ LRG1 can directly bind to the transforming growth factor‐β (TGF‐β) accessory receptor endoglin, which leads to activation of the proangiogenic Smad1/5/8 signaling pathway in endothelial cells and to pathogenic angiogenesis.^[^ ^11^ ^]^ In addition, increased expression of LRG1 has been observed in several types of carcinomas, including pancreatic, bladder, ovarian, and biliary tract cancer.^[^ ^13^ ^]^ During the initiation and progression of CRC, overexpression of LRG1 not only induces tumor angiogenesis but also promotes epithelial‐to‐mesenchymal transition (EMT) and the proliferation of CRC cells.^[^ ^14^ ^]^ Although overexpression of LRG1 is a prominent feature of various cancers, the dysregulation of its levels remains insufficiently understood.

Here, we showed that ELK4 promotes tumorigenesis and tumor progression in CRC in vitro and in vivo. Integrated genomics‐ and proteomics‐based approaches (ChIP‐seq, RNA‐seq, and IP‐MS analysis) identified the transcription factors SP1 and SP3 as novel coregulators of ELK4 at the genome‐wide level in CRC, instead of SRF, the well‐known cofactor of ELK4. LRG1 was found to be a direct target of the ELK4‐SP1/3 complex, which mediated the protumor angiogenic function of ELK4 in CRC. In response to serum stimulation, the interactions of ELK4 with SP1 and SP3 were found to be enhanced. Targeting the ELK4‐SP1/3 complex by combination treatment with a MEK/ERK inhibitor and an SP1 inhibitor elicited a synergistic antitumor effect. In addition to finding that ELK4 overexpression was a biomarker of poor prognosis in CRC patients, we developed a nine‐gene prognostic signature derived from the ELK4‐SP1/3 complex‐regulated gene set (ESGS) to predict CRC patient outcomes. Thus, our study reveals a new cooperative regulatory mechanism by which SP1/3 mediates ELK4 transcriptional activity in a noncanonical SP1/3‐dependent manner, emphasizing that the ELK4‐SP1/3 complex could be a potential therapeutic target for CRC.

## 2 Results

### 2.1 ELK4 Promotes the Malignant growth and Metastasis of Colorectal Cancer In Vitro and In Vivo

**Figure 1:**
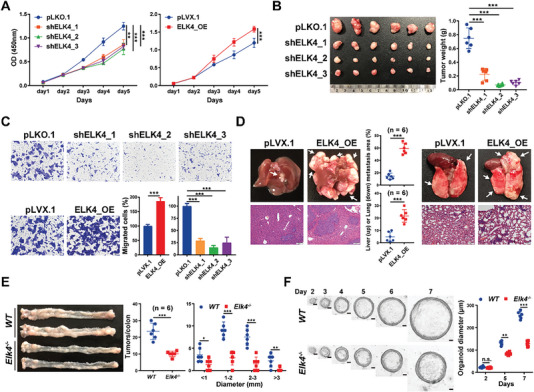
ELK4 is required for CRC cell proliferation and migration in vitro and for CRC tumor growth and metastasis in vivo. A,C) CCK8 A) and Transwell assays C) of HCT116 cells with ELK4 knockdown or overexpression. B) Representative images and statistical analysis of ELK4 knockdown HCT116 xenograft tumors (*n* = 6). D) Representative images of liver and lung metastases and hematoxylin‐eosin staining. The arrow indicates the metastatic loci. The tumor/hematoxylin‐eosin‐stained field was used for statistical analysis. E) Representative images of colon tumors from *WT* and *Elk4^−/−^* mice after AOM/DSS model induction. Tumor numbers and sizes were statistically analyzed (*n* = 6 mice/group), and each symbol represents an individual mouse. F) Representative images and quantification of the size of organoids formed from *WT* and *Elk4^−/−^* large intestine stem cells (scale bars = 20 µm). Two‐way ANOVA A), One‐way ANOVA B,C) and Student's *t* test C–F) were performed to assess the statistical significance. The data are presented as the mean ± S.D. values. * *P* < 0.05, ** *P* < 0.01, *** *P* < 0.001.

To explore the potential protumorigenic function of ELK4 in CRC, we generated HCT116 and LoVo cells with ELK4 overexpression or stable knockdown (Figure S1A,B, Supporting Information). As determined by CCK8 assays, knockdown of ELK4 significantly impaired the proliferation of HCT116 and LoVo cells, while ELK4‐overexpression promoted cell proliferation in vitro (**Figure** **1**A; Figure S1C, Supporting Information). Consistent with the in vitro assay results, knockdown of ELK4 suppressed tumor growth in vivo, as indicated by the decreased size and weight of ELK4 knockdown xenograft tumors compared with control group tumors and the decreased number of Ki67‐positive cells in ELK4 knockdown xenograft tumors (Figure 1B; Figure S1E, Supporting Information); in contrast, ELK4‐overexpressing promoted tumor growth in vivo (Figure S1D,E, Supporting Information).

In addition, the cell migration assays demonstrated that ELK4 overexpression enhanced the migration ability of HCT116 cells, whereas ELK4 knockdown elicited the opposite effect (Figure 1C). Similar results were observed in LoVo cells (Figure S1F, Supporting Information). Next, to explore the prometastatic function of ELK4 in vivo, we established two mouse models of metastasis. Markedly increased tumor burdens in the lungs and liver were validated by hematoxylin and eosin (H&E) staining in mice injected with ELK4‐overexpressing cells, suggesting that overexpression of ELK4 enhances tumor metastasis in CRC (Figure 1D).

Next, to explore the oncogenic role of ELK4 in CRC tumorigenesis, we established a model of azoxymethane‐dextran sodium sulfate (AOM‐DSS)‐induced colorectal tumorigenesis in wild‐type and *Elk4^−/−^* mice (Figure S1G, Supporting Information). We found that compared with their wild‐type littermates, *Elk4^−/−^* mice exhibited significant decreases in the number and size of colon tumors after AOM‐DSS administration (Figure 1E). The proliferation of tumor cells was also dramatically reduced in *Elk4^−/−^* mice (Figure S1H, Supporting Information). As shown in Figure 1F, organoids derived from *Elk4^−/−^* mice grew more slowly and had a smaller size in ex vivo culture, with reduced organoid numbers and shorter diameters than organoids derived from wild‐type mice. Taken together, these data suggest that overexpression of ELK4 can promote tumor growth and metastasis in CRC.

### 2.2 ELK4 Enhances Tumor Angiogenesis in CRC

**Figure 2:**
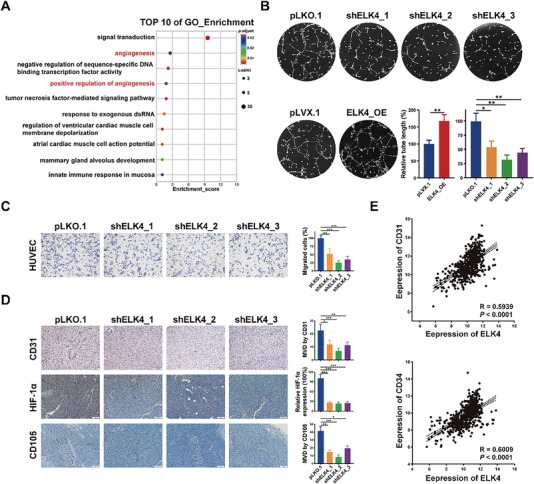
ELK4 promotes tumor angiogenesis in CRC. A) GO enrichment analysis of differentially expressed genes identified by RNA‐seq in ELK4 knockdown HCT116 cells. B,C) Representative images and statistical analysis of tube formation B) and Transwell assays C) of HUVECs in the presence of CM from HCT116 cells with ELK4 knockdown or overexpression. D) Representative images and statistical analysis of IHC staining for CD31, HIF‐1α, and CD105 in xenografts derived from pLKO.1‐ and shELK4‐transduced HCT116 cells. E) Correlation data between the mRNA level of ELK4 and those of CD31 and CD34 in the TCGA CRC dataset. Pearson correlation analysis was used to evaluate the associations. One‐way ANOVA B–D) and Student's *t* test B) were performed to assess the statistical significance. The data are presented as the mean ± S.D. values. * *P* < 0.05, ** *P* < 0.01, *** *P* < 0.001.

To explore the transcriptional target genes of ELK4 in CRC cells, RNA‐seq analysis was performed on ELK4 knockdown and control HCT116 cells (Figure S2A and Table S1, Supporting Information). Interestingly, Gene Ontology (GO) enrichment analysis of the differentially expressed genes showed that two of the top ten enriched biological processes were related to angiogenesis (**Figure** **2**A; Table S2, Supporting Information). The differentially expressed genes were enriched in “angiogenesis”, which included “HOXA3, SRPX2, ID1, CALCRL, FGF1, MCAM, and ANGPT2”, and “positive regulation of angiogenesis”, which included “LRG1, THBS1, FGF1, ANGPT2, and TWIST1”. First, we confirmed the downregulation of LRG1, HOXA3, SRPX2, and MCAM in three CRC cell lines (Figure S2B, Supporting Information). Of note, except for HOXA3, TWIST1, and ID1, the other seven genes are all proangiogenic genes that can be expressed and secreted by tumor cells, which implied the potential role of ELK4 in tumor angiogenesis in a paracrine manner in CRC. Next, to validate this hypothesis, we used an in vitro endothelial tube formation assay. Conditioned medium (CM) derived from cells with ELK4 overexpression stimulated greater tube formation of HUVECs, while CM from cells with ELK4 knockdown elicited lower tube formation (Figure 2B; Figure S2C, Supporting Information). Consistent with these results, Transwell assays of HUVECs demonstrated that CM derived from ELK4 knockdown cells impaired the migration ability of HUVECs, whereas CM from cells with ELK4 overexpression promoted the migration of HUVECs (Figure 2C; Figure S2D, Supporting Information). Furthermore, we examined the expression levels of two tumor vasculature markers, CD31 and CD105, in xenograft tumors formed by CRC cells with stable knockdown or overexpression of ELK4 by IHC staining to confirm the role of ELK4 in tumor angiogenesis in vivo. As shown in Figure 2D, knockdown of ELK4 led to a reduced microvessel density in xenograft tumors. Conversely, a significantly increased number of microvessels was observed in ELK4‐overexpressing tumors compared to control tumors (Figure S2E, Supporting Information). Notably, the protein level of the tumor hypoxia marker HIF1A in control xenograft tumors was higher than that in ELK4 knockdown xenograft tumors (Figure 2D), which could be due to the downregulation of HIF1A transcription by decreased expression of LRG1.^[^ ^14^, ^15^, ^16^ ^]^ This reflected the decreased tumor growth of ELK4 knockdown xenograft tumors, which could be due to attenuated tumor angiogenesis. Finally, a positive correlation between the expression of ELK4 and that of CD31 or CD34 was observed in the TCGA CRC dataset (*N* = 571) (Figure 2E). Collectively, these data corroborate the crucial positive role of ELK4 in tumor angiogenesis in CRC.

### 2.3 Genome‐Wide Screening and Functional Analysis Identify SP1/3 as the Key Coregulators of ELK4 in CRC

**Figure 3:**
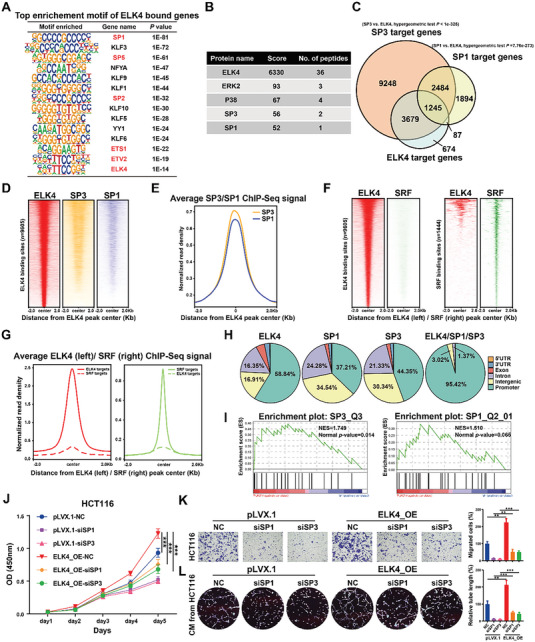
SP1/3, instead of SRF, are coregulators of ELK4 in CRC. A) Motif enrichment analysis of the most significant ELK4‐bound genes in HCT116 cells. B) Mass spectrometry (MS) identification of ELK4‐interacting proteins in HEK293T cells. C) Venn diagram displaying the overlap between ELK4, SP1, and SP3 target genes in HCT116 cells. D) Heatmaps of ChIP‐seq data for ELK4, SP1, and SP3 in HCT116 cells. All peaks in each heatmap are centered ± 2.0 kb from the ELK4 peaks in ELK4‐bound target genes. E) Normalized read density (per bp per peak) for ELK4, SP1, and SP3 plotted in the region ± 2.0 kb from the ELK4‐bound peaks. F) Heatmaps of ChIP‐seq data for ELK4 and SRF peaks in the ELK4 (left) and SRF (right) targets. G) Normalized read density (per bp per peak) for ELK4 and SRF plotted in the region ± 2.0 kb from the ELK4‐bound (left) or SRF‐bound (right) peaks. H) Pie charts displaying the distribution of genomic features bound by only ELK4, SP1, or SP3 and those bound by ELK4‐SP1/SP3. I) Gene set enrichment analysis (GSEA) of RNA‐seq data revealed that the SP1 and SP3 signatures were enriched upon knockdown of ELK4 in HCT116 cells. J,K) ELK4‐overexpressing HCT116 cells were transfected with control, SP1, and SP3 siRNAs for 2 days and were then subjected to CCK8 J) and Transwell K) assays. L) Representative images and statistical analysis of HUVEC tube formation incubated with CM from ELK4‐overexpressing HCT116 cells transfected with control, SP1, and SP3 siRNAs. Two‐way ANOVA J) and One‐way ANOVA (K and L) were performed to assess the statistical significance. The data are presented as the mean ± S.D. values. ** *P* < 0.01, *** *P* < 0.001.

In addition to performing RNA‐seq analysis, we also generated an ELK4 genomic binding profile in HCT116 cells to identify the direct target genes of ELK4 and its related transcriptional coregulators at the genome‐wide level. Peak annotation revealed that 5685 genes were associated with at least one ELK4 binding peak in HCT116 cells. As a TCF family member, ELK4 is well known to regulate target gene transcription by cooperating with the transcription factor SRF. Unexpectedly, motif enrichment analysis showed that the ELK4‐bound peaks in HCT116 cells were highly enriched with motifs of KLF/SP transcription factor family members, such as SP1, KLF3, and SP5, but not with the SRF motif. These data indicate that ELK4 may functionally interact with KLF/SP transcription factors but not the well‐known coregulator SRF in CRC cells (**Figure** **3**A). We also performed IP‐MS/MS to identify the ELK4 binding proteins in HCT116 cells. Notably, as revealed by IP‐MS/MS, only the KLF/SP transcription factor family members SP1 and SP3 were found to interact with ELK4 in HCT116 cells (Figure 3B). These results led us to hypothesize that SP1 and SP3 act as the key transcriptional coregulators of ELK4 in CRC.

To further test this hypothesis, we obtained published SP1 ChIP‐seq data in HCT116 cells (GSM1010902)^[^ ^17^ ^]^ and SP3 ChIP‐seq data in HEK293 cells (GSE91528)^[^ ^18^ ^]^ to test whether ELK4/SP1 and ELK4/SP3 occupy shared genomic regions. The intersection of the sets of ELK4 target genes and SP1‐ or SP3‐associated genes revealed that >85% of ELK4 target genes were also occupied by SP1 or SP3 (significant overlap validated by hypergeometric test, *P* = 7.7e‐273 and < 1e‐325 for SP1 and SP3, respectively) (Figure 3C). KEGG analysis of these overlapping target genes identified significant enrichment of the AMPK, Wnt, and Hippo signaling pathways, which are known to be associated with tumorigenesis (Figure S3A, Supporting Information). Importantly, genomic landscape analysis showed that SP1 and SP3 were significantly enriched in regions surrounding ELK4 binding sites (Figure 3D,E). Mapping of the distances between binding sites further revealed that 73% of the shared target genes had SP3 and ELK4 binding sites within 1 kb of each other and that 37% of the shared target genes had SP1 and ELK4 binding sites within 1 kb of each other (Figure S3B, Supporting Information). Next, we compared the ELK4 genomic binding profile with the public ChIP‐seq data of SRF in HCT116 cells (GSM1010851).^[^ ^17^ ^]^ Consistent with the results of the motif enrichment analysis, the majority of ELK4 targets were bound by ELK4 but not SRF. In addition, the majority of SRF targets were bound by SRF but not ELK4 (Figure 3F,G). These data further supported the hypothesis that ELK4 may not cooperate with SRF in CRC cells. As shown in Figure S3C (Supporting Information), the oncogenes MFSD12 and VCP were bound by ELK4, SP1, and SP3 at the gene promoters. Furthermore, the classification of binding sites with respect to the locations of genes revealed that a large fraction of the sites with shared ELK4‐SP1 or ELK4‐SP3 occupancy were in gene promoters (95.42%), while the majority of ELK4, SP1, and SP3 binding sites were generally distributed across different genomic regions (Figure 3H). We further reanalyzed the ChIP‐seq data by focusing on the ELK4‐bound promoters (Figure S3D–H, Supporting Information). Consistently, these data suggested that ELK4 and SP1 or SP3 occupy the same promoters and form cis‐regulatory modules to transcriptionally regulate target gene expression in CRC (Figure S3D–H, Supporting Information). In support of this idea, gene set enrichment analysis (GSEA) showed that the SP1 and SP3 gene signatures were suppressed in ELK4 knockdown cells (Figure 3I).

Next, to further study whether the oncogenic transcription factors SP1/3 functionally cooperate with ELK4 in CRC, SP1, and SP3 were knocked down in HCT116 and LoVo cells stably overexpressing ELK4. We observed that ELK4 overexpression‐induced cell proliferation, migration, and angiogenesis were significantly attenuated by SP1 or SP3 knockdown (Figure 3J–L; Figure S3I–K, Supporting Information). Altogether, our results demonstrate that both SP1 and SP3 could be the key transcriptional coregulators of ELK4 in CRC and that the ELK4‐SP1/3 transcriptional complex promotes the progression of CRC.

### 2.4 Serum Stimulation Enhances the Interactions between ELK4 and SP1/3

**Figure 4:**
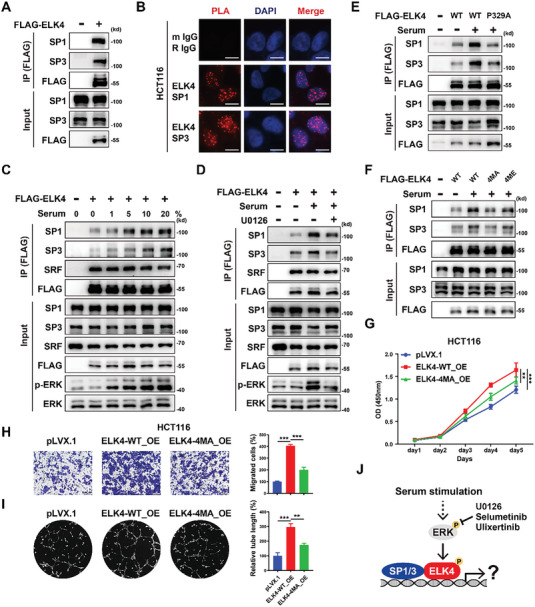
Serum stimulation‐mediated phosphorylation of ELK4 facilitates its interaction with SP1 and SP3. A) Coimmunoprecipitation (Co‐IP) to evaluate the interactions between exogenous FLAG‐ELK4 and endogenous SP1 and SP3 in HCT116 cells. B) Proximity ligation assay (PLA) to evaluate the interactions of ELK4 with SP1 and SP3 in HCT116 cells. The red dots indicate protein interactions (scale bars = 10 µm). C) Serum stimulation enhances the interactions between ELK4 and SP1 and SP3 in HCT116 cells. The indicated plasmids were transfected, and cells were then treated with the indicated serum concentrations for 15 min. D) U0126 suppresses the serum‐induced interactions between ELK4 and SP1 and SP3 in HCT116 cells. HCT116 cells were transfected with the indicated plasmids and were then cultured in the presence or absence of U0126 (10 µm) followed by stimulation with 10% serum for 15 min. E) HCT116 cells were transfected with ELK4 wild‐type and P329A mutant plasmids for 24 h prior to stimulation with 10% serum for 15 min. F) HCT116 cells were transfected with wild‐type ELK4 and various ELK4 mutant plasmids (4MA: T361/T366/S381/S387A, 4ME: T361/T366/S381/S387E) followed by stimulation with 10% serum for 15 min. G–I) HCT116 cells overexpressing WT ELK4 or 4MA mutant ELK4 were subjected to CCK8 G), Transwell H) and tube formation I) assays. J) Schematic illustration of serum stimulation‐induced phosphorylation of ELK4 to enhance its interaction with SP1 and SP3; the transcriptional regulatory mechanism of the ELK4‐SP1/3 complex is still unknown. Two‐way ANOVA G) and One‐way ANOVA H,I) were performed to assess the statistical significance. The data are presented as the mean ± S.D. values. ** *P* < 0.01, *** *P* < 0.001.

IP‐MS/MS analysis of HCT116 cells expressing FLAG‐tagged ELK4 identified SP1 and SP3 as the KLF/SP transcription factor family members that interact with ELK4 in HCT116 cells. In addition, the known kinases mediating ELK4 phosphorylation, including ERK2 and p38, were identified in HCT116 cells (Figure 3B). First, a reciprocal coimmunoprecipitation assay confirmed that ELK4 can interact with both SP1 and SP3 (**Figure** **4**A; Figure S4A, Supporting Information). Furthermore, the proximity ligation assay (PLA) showed the interactions between endogenous ELK4 and endogenous SP1 and SP3 in HCT116, SW480, and RKO cells (Figure 4B; Figure S4B, Supporting Information). Phosphorylation of ETS proteins by distinct classes of MAPKs, including ERK, JNK, and p38, can alter transcriptional functions via various mechanisms. We then sought to determine whether serum‐induced MAPK signaling can affect the interactions between ELK4 and SP1/3. Intriguingly, serum stimulation enhanced the interactions between ELK4 and SP1/3 in a dose‐dependent manner (Figure 4C). Pretreatment with the MEK inhibitors U0126 and selumetinib or the ERK inhibitor ulixertinib markedly decreased the serum‐induced interactions between ELK4 and SP1/3 (Figure 4D; Figure S4C–E, Supporting Information). Previous studies have reported that substitution of the ELK4 residue Pro329 with Ala (P329A) severely disrupts the interaction between ELK4 and ERK2.^[^ ^19^ ^]^ Compared with wild‐type ELK4, P329A mutant ELK4 exhibited decreased associations with SP1/3 (Figure 4E). Furthermore, in vitro dephosphorylation by λ‐protein phosphatase (λ‐PPase) almost completely abolished the serum‐induced ELK4‐SP1/3 interaction, which suggested that phosphorylation is critical for the ELK4‐SP1/3 interaction (Figure S4F, Supporting Information). Given that ELK4 is phosphorylated by ERKs at Thr361, Thr366, Ser381, and Ser387,^[^ ^19^ ^]^ we also generated serial ELK4 mutants in which these four phosphorylation sites were replaced with alanine. The T361/T366/S381/S387A mutation but not the phosphomimetic T361/T366/S381/S387E mutation abolished the serum‐induced enhancement in the interactions between ELK4 and SP1/3 (Figure 4F). In addition, the interaction between T361/T366/S381/S387E ELK4 and SP1 was more stable than that between WT ELK4‐SP1 with U0126 treatment (Figure S4G, Supporting Information). Deletion of C‐terminal (Δ352‐402) ELK4 largely disrupted the interaction between ELK4 and SP1/3, which further supported that the C‐terminal phosphorylation sites of ELK4 are required for the ELK4‐SP1/3 interaction (Figure S4H, Supporting Information).

Next, we tried to map the domains of SP1/3 mediating the interaction with ELK4. We generated serial truncations of SP1 and SP3 with deletion of different protein domains (Figure S5A,B, Supporting Information). Interestingly, we found that deletion of the D domain of SP1 diminished the association of SP1 with ELK4, while loss of the zinc finger domain of SP3 led to an attenuated interaction of SP3 with ELK4 (Figure S5A,B, Supporting Information). However, deletion of the SP1 D domain or the SP3 ZNF domain resulted in cytoplasmic translocation of SP1 and SP3. Thus, the attenuated ELK4‐SP1‐∆D and ELK4‐SP3‐∆ZNF might be an indirect consequence of cytoplasmic translocation of truncated SP1 and SP3 (Figure S5C, Supporting Information). It has been reported that ERK can phosphorylate SP1 to promote its nuclear translocation.^[^ ^20^ ^]^ However, we did not observe the subcellular translocation of SP1 upon serum stimulation and U0126 treatment in CRC cells (Figure S5D, Supporting Information), which further suggested that the diminished ELK4‐SP1 interaction by MEK‐ERK inhibition was not due to the reduced level of nuclear SP1 protein. Finally, we explored whether phosphorylation of ELK4 (Thr361, Thr366, Ser381, and Ser387) affects its oncogenic functions. As expected, we found that the mutation of T361/T366/S381/S387A significantly abrogated the protumorigenic function of ELK4 in cell proliferation, cell migration, and tumor angiogenesis (Figure 4G–I). Overall, our data indicate that serum stimulation‐induced phosphorylation of ELK4 facilitates its interaction with SP1 and SP3 which could be vital for ELK4's oncogenic function in CRC (Figure 4J).

### 2.5 ELK4 and SP1/3 Cooperatively Activate LRG1 Transcription to Promote Tumor Angiogenesis in CRC

**Figure 5:**
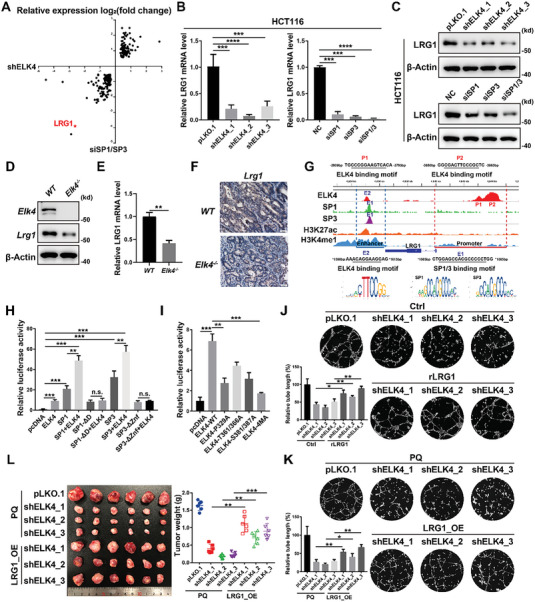
LRG1 is the direct target of the ELK4‐SP1/3 complex in CRC. A) Scatter plot showing that a set of genes was differentially expressed by both ELK4 knockdown and SP1/3 knockdown in HCT116 cells (*P* < 0.05). B,C) qPCR B) and western blot C) analyses of the LRG1 mRNA level in HCT116 cells with ELK4 knockdown or SP1/3 knockdown. D–F) Detection of the expression of ELK4 and LRG1 in colorectal cancer tissues derived from *WT* and *Elk4^−/−^* mice by western blot D), qPCR E), and IHC F) analyses. G) Schematic depiction of the LRG1 gene locus showing the potential LRG1 enhancer and promoter, as identified by ChIP‐seq data of H3K27ac (GSM2058026) and H3K4me1 (GSM2712765). The ELK4 (red), SP1 (green), SP3 (purple), H3K27ac (orange), and H3K4me1 (blue) peaks and potential binding sites are shown. H) Luciferase assay of HCT116 cells cotransfected with the LRG1 reporter containing the promoter and enhancer and empty vector or the indicated constructs of ELK4, SP1, and SP3. I) Luciferase assay of HCT116 cells cotransfected with the LRG1 reporter containing the promoter and enhancer and empty vector, the WT ELK4 plasmid, or mutant ELK4 plasmids. J,K) CM was collected from ELK4 knockdown HCT116 cells stimulated with 500 ng mL^−1^ rLRG1 J) or modified by LRG1 overexpression K) and was then applied to HUVECs for the tube formation assay. L) Representative images of xenograft tumors derived from ELK4 knockdown HCT116 cells overexpressing LRG1 are shown (left); xenografts were weighed for statistical quantification (right). Student's *t* test E), and One‐way ANOVA B, H–L) were performed to assess the statistical significance. The data are presented as the mean ± S.D. values. * *P* < 0.05, ** *P* < 0.01, *** *P* < 0.001.

To identify the direct target gene cooperatively regulated by ELK4 and SP1/3 to enhance tumor angiogenesis, we also generated the gene transcription profiles of SP1/3 knockdown HCT116 cells by RNA‐seq and found a marked overlap of genes that were differentially expressed by ELK4 knockdown or SP1/3 knockdown (*P* < 0.05) (**Figure** **5**A). First, we confirmed that the mRNA levels of LRG1, STC2, and LBHD1 were downregulated in both ELK4‐ and SP1/3‐knockdown HCT116, SW480, and RKO cells (Figure S6A, Supporting Information). By integrating the RNA‐seq and ChIP‐seq data, we further performed GO analysis based on the newly identified ELK4‐SP1/3 coregulated genes. Consistently, the ELK4‐SP1/3 coregulated genes were also enriched in biological processes related to angiogenesis (Figure S6B, Supporting Information). Next, we were prompted to further investigate the LRG1 gene, since LRG1 was one of the angiogenesis‐related genes most strongly downregulated by knockdown of both ELK4 and SP1/3 and was also cobound by ELK4 and SP1/3 (Figure 5A,G). We first confirmed the decreased mRNA and protein levels of LRG1 in HCT116 and LoVo cells with ELK4 or SP1/3 knockdown (Figure 5B,C; Figure S6C, Supporting Information). The secreted LRG1 protein levels were also significantly decreased in ELK4‐ or SP1/3‐knockdown HCT116 cells (Figure S6D, Supporting Information). In contrast, overexpression of ELK4 increased the expression of LRG1 in HCT116 and LoVo cells (Figure S6C,E, Supporting Information). To further verify the ELK4‐LRG1 regulatory axis in vivo, we examined the expression of LRG1 in tumor tissues derived from wild‐type and *Elk4^−/−^* mice and from ELK4 OE/KD xenograft tumors (Figure S6F, Supporting Information). Consistent with the observation in CRC cells in vitro, both the mRNA and protein levels of LRG1 were decreased in tumors derived from *Elk4^−/−^* mice (Figure 5D‐F). Next, we explored the molecular mechanism by which the ELK4‐SP1/3 complex regulates LRG1 transcription. The ChIP‐seq data revealed that ELK4, SP1, and SP3 occupy an enhancer (marked with high H3K27ac and H3K4me1 signals) located 1.4 kb downstream of the LRG1 gene, which was confirmed by ChIP‐qPCR in HCT116 cells (Figure 5G; Figure S7A, Supporting Information). In addition, there was a broad peak of ELK4 at the promoter of LRG1, located 3 kb to 4 kb upstream of the LRG1 TSS (Figure 5G; Figure S7A, Supporting Information). We therefore constructed two luciferase reporter plasmids: one harbored only the downstream enhancer, and the other harbored both the downstream enhancer and upstream promoter of ELK4 (Figure S7B,C, Supporting Information). As expected, overexpression of ELK4 or SP1/3 alone increased the luciferase activity of the downstream enhancer reporter, while coexpression of ELK4 with SP1 or SP3 showed a synergistic effect (Figure S7D, Supporting Information). Similar results were observed for the enhancer+promoter reporter (Figure S7D, Supporting Information). Interestingly, the enhancer+promoter reporter showed higher luciferase activity than the enhancer‐only reporter, indicating that upstream ELK4 binding also contributes to the transcriptional activation of LRG1 by ELK4 (Figure S7D, Supporting Information). We further constructed an enhancer+promoter luciferase reporter with mutation of ELK4 binding sites or SP1/3 binding sites and found that either mutation of ELK4 binding sites or SP1/3 binding sites diminished the activation of the enhancer+promoter luciferase reporter by overexpression of ELK4 or SP1/3 (Figure S7E, Supporting Information). In addition, we observed that the SP1/3 truncations lost the ability to coactivate the LRG1 enhancer+promoter reporter in cooperation with ELK4 (Figure 5H). Furthermore, the ERK binding‐defect P329A mutant of ELK4 and the phosphorylation‐defective mutants of ELK4 showed attenuated activation of the LRG1 enhancer+promoter reporter (Figure 5I). These data demonstrate that the interaction between ELK4 and SP1/3 is essential for the transcriptional activation of LRG1 by the ELK4‐SP1/3 complex.

Next, we explored whether LRG1 mediates the proangiogenic role of the ELK4‐SP1/3 complex in CRC. To elucidate this point, recombinant LRG1 protein was added to conditioned medium derived from ELK4 knockdown HCT116 cells, and we observed that recombinant LRG1 protein partially rescued the decreased stimulation of tube formation (Figure 5J). Furthermore, a similar result was observed in ELK4 knockdown HCT116 cells overexpressing LRG1: the loss of enhanced stimulation of tube formation by CM from ELK4 knockdown cells was partially reversed by overexpression of LRG1 (Figure 5K; Figure S7F, Supporting Information). LRG1 has been shown to promote angiogenesis by modulating endothelial TGF‐β signaling and the subsequent phosphorylation of SMAD1/5. We observed that conditioned medium derived from ELK4 knockdown HCT116 cells lost the ability to promote phosphorylation of SMAD1/5 in HUVECs, while either rescued expression of LRG1 or recombinant LRG1 protein restored the phosphorylation of SMAD1/5 in HUVECs (Figure S7G,H, Supporting Information). In addition, conditioned medium derived from ELK4‐overexpressing HCT116 cells enhanced the phosphorylation of SMAD1/5 in HUVECs, which was partially abrogated by knockdown of LRG1 (Figure S7G, Supporting Information). Consistent with these results, overexpression of LRG1 significantly blunted the decreased growth of ELK4 knockdown HCT116 xenografts (Figure 5L). Immunostaining showed a significant increase in microvessels (Figure S7I, Supporting Information). Collectively, our data demonstrate that LRG1 is transcriptionally regulated by the ELK4‐SP1/3 complex and is required for the oncogenic function of the ELK4‐SP1/3 complex, especially in tumor angiogenesis.

### 2.6 U0126 and Mithramycin a Synergistically Suppress CRC Growth

**Figure 6:**
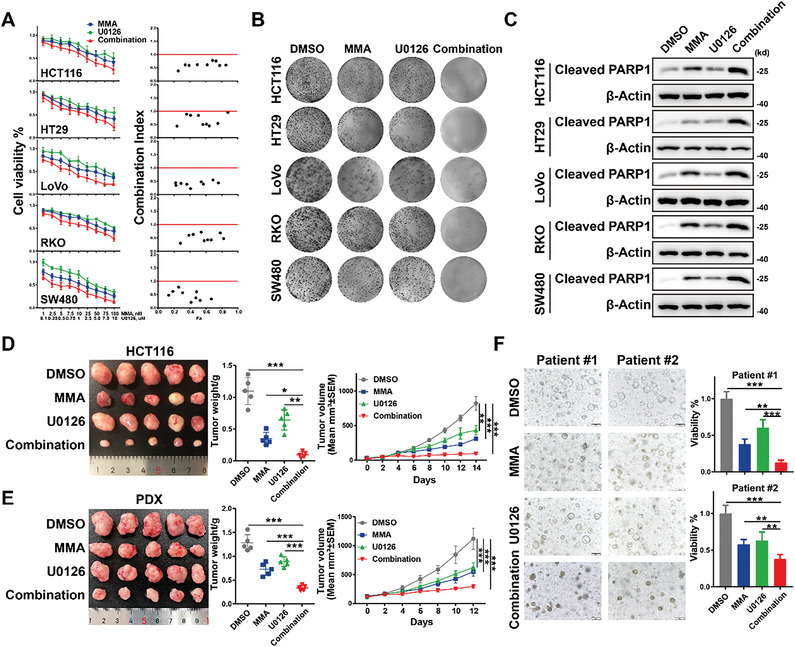
Combination treatment with a MEK/ERK inhibitor (U0126) and an SP1 inhibitor (mithramycin A, MMA) has potent antitumor activity in CRC. A) Cell proliferation was assessed following 48 h of exposure to the indicated concentrations of MMA, U0126, or their combination in HCT116, HT29, LoVo, RKO, and SW480 cells. CI values for the various combinations were calculated using CompuSyn. A CI < 1.0 indicates a synergistic effect. B) Colony formation assays showed a synergistic response to the combination of U0126 and MMA in HCT116, HT29, LoVo, RKO, and SW480 cells. C) Representative western blots of cleaved PARP1 in HCT116, HT29, LoVo, RKO, and SW480 cells treated with vehicle, 50 nm MMA, 5 µm U0126, or the combination of MMA and U0126. D,E) Representative images of HCT116 cell‐derived D) and CRC patient‐derived E) xenografts harvested from nude mice treated with vehicle, 0.5 mg kg^−1^ MMA, 10 mg kg^−1^ U0126 or the combination of MMA and U0126 (*n* = 5 mice per group) (left). The tumors were weighed (middle), and growth curves were plotted (right). F) Representative images of two PDOs treated with vehicle, 50 nm MMA, 5 µm U0126 or the combination of MMA and U0126 for 48 h (left); cell viability was evaluated with a CellTiter‐Glo assay (right). One‐way ANOVA D–F) and Two‐way ANOVA D,E) were performed to assess the statistical significance. The data are presented as the mean ± S.D. or mean ± S.E.M. values. ** *P* < 0.01, *** *P* < 0.001.

Our finding that the ELK4‐SP1/3 complex cooperatively regulates gene expression to promote CRC tumor growth raises the intriguing possibility that the combination of a MEK/ERK inhibitor with an SP1 inhibitor might lead to a synergistic inhibitory effect on CRC growth. To test this hypothesis, five CRC cell lines were treated with U0126, a MEK/ERK inhibitor, and mithramycin A (MMA), an SP1 inhibitor that not only attenuates the binding of SP1 to DNA but also promotes the proteasome‐dependent degradation of SP1 (Figure S8A, Supporting Information). Western blot analysis confirmed the inhibitory effect of U0126 and MMA on ERK and SP1, respectively (Figure S8B, Supporting Information). Intriguingly, the protein level of LRG1 decreased more in CRC cells treated with the combination of U0126 and MMA than in CRC cells treated with a single agent, which implied the synergistic inhibitory effect of U0126 and MMA (Figure S8B, Supporting Information). Consistently, CCK8 and colony formation assays showed that compared to treatment with either single agent, cotreatment of CRC cells with U0126 and MMA exhibited a significantly enhanced inhibitory effect on cell proliferation (**Figure** **6**A,B). The combination index (CI) of each combination treatment, calculated via the Chou‐Talalay method, was less than 1, which indicated the synergistic antitumor activity of the U0126/MMA combination (Figure 6A). Western blot analysis of the apoptosis marker cleaved PARP1 further demonstrated that cotreatment with U0126/MMA induced more cell apoptosis than single agent treatment (Figure 6C). Similar results were observed by using the FDA‐approved MEK inhibitor selumetinib and MMA (Figure S8C–E, Supporting Information). Next, to rule out the possibility of off‐target effects of MMA, we further examined the synergistic effect of U0126 and SP1 knockdown in HCT116 cells. We observed that knockdown of SP1 induced moderately enhanced levels of cleaved PARP1 in HCT116 cells and further increased the level of cleaved PARP1 in HCT116 cells treated with U0126, which showed the synergistic effect of U0126 and SP1 knockdown (Figure S8F, Supporting Information). More importantly, MMA/U0126 cotreatment did not induce more cell apoptosis than a single agent in SP1 knockdown HCT116 cells (Figure S8F, Supporting Information). These data suggest that the synergistic effect of MMA/U0126 cotreatment relies on the inhibition of SP1 by MMA.

Subsequently, to determine the potential synergistic effect of combination treatment with U0126 and MMA in vivo, xenograft assays were carried out in both cell‐based (CDX) and patient‐derived xenograft (PDX) models. Treatment with the single agents U0126 and MMA or combination treatment did not show observable cytotoxicity in vivo (Figure S9A, Supporting Information). Western blot analysis further confirmed the decreased phosphorylation level of ERK in the U0126‐treated groups and the downregulated protein level of SP1 in the MMA‐treated groups (Figure S9B, Supporting Information). As expected, we observed that in both the CDX and PDX models, tumor growth was significantly suppressed by treatment with the combination of U0126 and MMA compared with treatment with either single agent (Figure 6D,E). IHC staining in HCT116 xenografts further revealed a dramatically decreased number of proliferating Ki67‐positive cancer cells and an increased number of apoptotic cancer cells in the U0126/MMA combination group (Figure S9C, Supporting Information). Combination treatment with U0126 and MMA showed a synergistic suppressive effect on LRG1 expression both in vitro and in vivo (Figures S8B and S9B, Supporting Information). Thus, we further assessed the effect of combination treatment with U0126 and MMA in LRG1‐overexpressing HCT116 cells in vivo. Overexpression of LRG1 dramatically restored tumor growth after U0126/MMA cotreatment, which suggested that synergistic downregulation of LRG1 mediated the antitumor effect of combination treatment (Figure S9D, Supporting Information). To further explore the translational therapeutic potential of combination treatment with U0126 and MMA, two patient‐derived organoid (PDO) models were established. Consistent with the results of xenograft assays, treatment with U0126 or MMA alone resulted in a moderate decrease in the growth of PDOs, and the combined treatment resulted in the lowest cell viability in PDOs (Figure 6F). Overall, the data indicate that the combination of MEK/ERK inhibitors with SP1 inhibitors could be a promising combination drug treatment for CRC.

### 2.7 Clinical Implication of ELK4 Expression and Correlation of the ELK4/SP1/3‐LRG1 Axis in CRC

**Figure 7:**
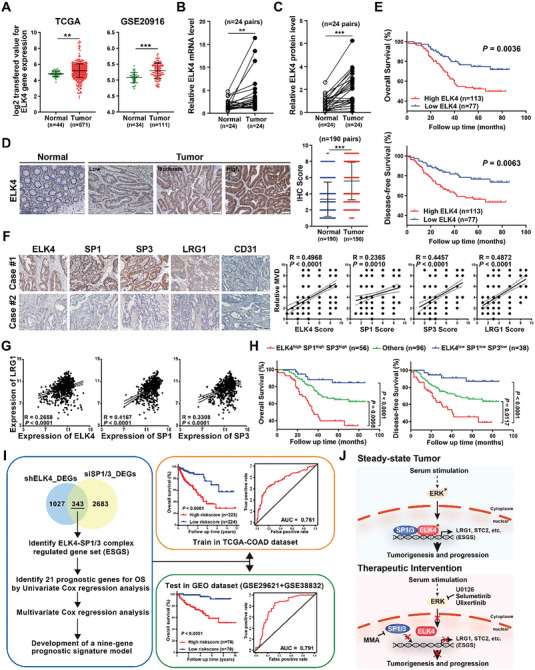
Association of ELK4 upregulation with poor prognosis and the clinical relevance of the ELK4/SP1/3‐LRG1 axis in CRC. A) Relative ELK4 mRNA levels in CRC tumor and normal tissues were analyzed based on the TCGA database (left) and GSE20196 (right). B,C) Relative mRNA B) and protein C) levels of ELK4 in 24 pairs of CRC and adjacent normal tissues. D) Representative images (left) and quantitative analysis (right) of 190 pairs of CRC tissues in the TMA cohort based on ELK4 IHC staining. E) Kaplan‒Meier plots of overall survival (up) and disease‐free survival (down) for 190 CRC patients stratified by the ELK4 expression level (data from the TMA). F) Positive correlations between relative CD31‐assessed microvessel density (MVD) value and ELK4, SP1, SP3, and LRG1 expression in the CRC TMA cohort. Representative images (left) and statistical analysis (right) of IHC staining for ELK4, SP1, SP3, and LRG1 in CRC tissue (scale bars = 50 µm). Pearson correlation analysis was used to evaluate the associations. G) Correlation data between LRG1 mRNA expression and ELK4, SP1, and SP3 mRNA expression in the TCGA CRC dataset. Pearson correlation analysis was used to evaluate the associations. H) Kaplan‒Meier analysis of overall survival (left) and disease‐free survival (right) in the TMA cohort according to the combined expression status of ELK4, SP1, and SP3. I) A schematic illustration of the development of an ESGS‐related prognostic model for CRC patients. J) A schematic illustration of our findings on the transcriptional regulatory mechanism and biological function of ELK4 in CRC. A paired Student's *t* test B,C) and Wilcoxon signed‐rank test D) were performed to assess the statistical significance. The data are presented as the mean ± S.D. values (Student's *t* test). ** *P* < 0.01, *** *P* < 0.001.

Finally, to determine the clinical relevance of dysregulated ELK4 in CRC, we analyzed the mRNA expression of ELK4 in the TCGA CRC dataset and GEO dataset (GSE20916) and demonstrated the increased mRNA expression level of ELK4 in CRC tissues (**Figure** **7**A). We confirmed the overexpression of ELK4 in CRC by qPCR and western blot analyses in 24 CRC tissues as well as paired adjacent normal tissues (Figure 7B,C; Figure S7A, Supporting Information). In addition, the ELK4 expression level was found to be higher in seven CRC cell lines than in two normal colonic epithelial cell lines (Figure S7B, Supporting Information). Next, we performed a large‐scale IHC analysis in CRC tissue arrays containing 190 paired CRC and matched adjacent tissues, which further confirmed that ELK4 was overexpressed in CRC (Figure 7D). Furthermore, Kaplan‐Meier analysis indicated that a higher ELK4 expression level was associated with poorer overall survival (OS) and disease‐free survival (DFS) in patients with CRC (Figure 7E). Similar results were obtained from analysis of the TCGA CRC dataset, in which a higher mRNA expression level of ELK4 was correlated with a shorter DFS time (Figure S7C, Supporting Information). These data suggest that upregulation of ELK4 is an indicator of poor prognosis in CRC.

To examine the robustness of the ELK4/SP1/3‐LRG1 axis in CRC, we assessed SP1, SP3, and LRG1 protein expression by IHC staining in the same CRC cohort. IHC analysis showed that the LRG1 protein level was positively correlated with the protein levels of ELK4, SP1, and SP3 in our CRC cohort (Figure 7F; Figure S7D, Supporting Information). The positive associations between LRG1 and ELK4/SP1/SP3 were further validated in the TCGA and GSE20916 cohorts at the mRNA level (Figure 7G; Figure S7E, Supporting Information). Elevated expression of SP1 and SP3 was found to be a marker of poor prognosis in CRC. Additionally, we observed that the patients classified as ELK4^high^/SP1^high^/SP3^high^ showed the worst disease outcomes (Figure 7H). Of note, we also observed a positive correlation between the tumor vasculature marker CD31 and the protein levels of LRG1, ELK4, SP1 and SP3 in our CRC cohort (Figure 7F). These data imply that the enhanced ELK4/SP1/3‐LRG1 axis could promote tumor angiogenesis in CRC.

Since tumor classification based on gene expression profiles/signatures has shown more accuracy than classification based on single biomarkers, we hypothesized that an ELK4‐SP1/3 complex‐regulated gene signature could more precisely predict the prognosis of CRC patients. Thus, we developed a gene expression prognostic model based on the ELK4‐SP1/3 complex‐regulated gene set (ESGS) (Figure 7I). The overlapping DEGs between cells with ELK4 knockdown and cells with SP1/3 knockdown were termed ESGSs (343 genes). Through univariate Cox regression analysis, 21 ELK4‐SP1/3 complex‐regulated genes with prognostic value were identified. Stepwise multivariate Cox regression analysis was performed to develop an optimal prognostic model for overall survival (OS), and an ESGS with 9 genes was established. The risk score was calculated based on the coefficients of the individual ELK4‐SP1/3 complex‐regulated genes as follows: (1.01116 × ASB6) + (0.27059 × SLC43A3) + (0.11043 × MAGEA3) + (0.63964 × C2orf68) + (0.24183 × STC2) + (−0.63955 × MRPS7) + (0.42629 × LBHD1) + (−0.92017 × WDR31) + (0.50553 × PPM1N). Then, all patients were separated into a high‐risk and a low‐risk group according to the median value of the risk score. In both groups, as the risk scores of patients increased, the number of deaths increased (Figure S10F,G, Supporting Information). Our ESGS‐based prognostic model resulted in an AUC of 0.761 in the training set (TCGA‐COAD) (Figure 7I). To further verify the prognostic value of the nine‐gene ESGS‐based prognostic model in the test set (GSE20916+GSE38832), the 157 patients in the test set were classified according to the risk score. The high‐risk group had a worse prognosis than the low‐risk group, and the AUC of the test set was 0.791 (Figure 7I). Overall, these data demonstrate that the ESGS‐related prognostic model could be used to independently predict OS in CRC patients, further supporting the oncogenic role of the ELK4‐SP1/3 transcriptional complex in CRC.

## 3 Discussion

ETS transcription factor family members are widely associated with solid tumors.^[^ ^21^ ^]^ ELK1, ELK3, and ELK4 comprise the TCF subfamily of ETS transcription factors and are characterized by the formation of complexes with SRF dimers on SREs found in the promoters of c‐fos and other IEGs.^[^ ^22^ ^]^ As transcriptional activators of the oncogene c‐fos, ELK1, and ELK4 are considered oncogenes in breast cancer and melanoma.^[^ ^7^, ^23^ ^]^ Previously, we revealed that the oncogene LAMB3 is transcriptionally activated by ELK4 in CRC, implying that ELK4 may be involved in CRC tumor progression.^[^ ^10^ ^]^ In this study, analysis of our cohort and public datasets indicated that overexpression of ELK4 in CRC is a marker of poor prognosis and that ELK4 is an important driver of tumorigenesis and tumor progression in CRC.

Both ELK1 and ELK4 are well known as transcriptional coregulators of SRF and are thought to function primarily by complexing with SRF.^[^ ^22^ ^]^ However, the expression of c‐fos, the well‐known direct target of ELK1/4 through its complex with SRF, was not affected by knockdown of ELK4 in CRC cells. This suggests that ELK4 may not cooperate with SRF to regulate gene transcription in CRC. Consistent with these results, we observed a loss of enrichment of the SRF binding motif in ELK4 binding peaks in CRC cells. Thus, these findings indicate that ELK4 can regulate gene expression in an SRF‐independent manner in CRC. Intriguingly, previous ChIP‐chip analysis of ELK1 binding profiles in HeLa cells also implied that a considerable proportion of ELK1 binding sites (more than 77%) are not occupied by SRF.^[^ ^24^ ^]^ A similar result indicated that a large fraction of ELK4 peaks do not overlap with SRF peaks in macrophages.^[^ ^25^ ^]^ Similar to the situation for SRF, for which MRTF and TCFs regulate distinct transcriptional programs, it can be speculated that ELK4 exhibits both SRF‐dependent and SRF‐independent transcriptional profiles and biological functions in different cellular contexts or in response to different stimuli.

All ETS transcription factors possess highly conserved DNA binding ETS domains that recognize the common core DNA motif 5′‐GGA(A/T)−3′, yet they still vary in DNA binding specificity and regulate distinct target genes.^[^ ^22^ ^]^ The DNA binding specificity of each ETS member could be affected by many other DNA binding proteins that interact with ETS transcription factors.^[^ ^22^ ^]^ In this study, we identified the known oncogenic transcription factors SP1 and SP3, instead of SRF, as the primary functional partners of ELK4 at the genome‐wide level in CRC. SP1 has been reported to cooperate with another ETS transcription factor, ETS1, to transactivate the expression of target genes, such as PDGF‐A, PTHrP and DHX15.^[^ ^26^ ^]^ These previous studies focused on a specific target gene; however, we describe a comprehensive genome‐wide cooperative DNA binding and gene transcription regulation mechanism between ELK4 and SP1/3 in CRC. Notably, ELK4/SP1/SP3 mainly colocalize at gene promoters, although these three transcription factors generally bind across different genomic regions. In addition, since SP1 and SP3 recognize the same GC‐rich consensus binding motif,^[^ ^27^ ^]^ it is not surprising that both SP1 and SP3 can colocalize at the same regulatory elements with ELK4. However, we found that SP1 mainly interacts with ELK4 through its D domain, while SP3 interacts with ELK4 through its ZNF domain. The carboxy‐terminal D domain of SP1 is required for synergistic activation of SP1, and the ZNF domain of SP3 contains three Cys2His2 zinc fingers and is required for sequence‐specific DNA binding to GC boxes.^[^ ^27^ ^]^ These data imply the different cooperative regulatory mechanisms of the ELK4‐SP1 and ELK4‐SP3 protein complexes, which need to be explored in the future.

LRG1 is a newly identified regulator of pathogenic angiogenesis and tumor angiogenesis. In CRC, LRG1 not only promotes neovascularization but also promotes cell proliferation and induces EMT.^[^ ^14^, ^28^ ^]^ Knockout of Lrg1 or treatment with a blocking antibody against LRG1 significantly extended the survival of Apc^Min^ mice.^[^ ^29^ ^]^ The serum LRG1 protein level is increased in both ulcerative colitis (UC) and CRC.^[^ ^30^ ^]^ In addition, the increased mRNA level of LRG1 in CRC tissues compared with normal mucosal tissues implies that gene transcriptional activation could be one of the drivers of LRG1 upregulation in CRC.^[^ ^14^ ^]^ Furthermore, our findings demonstrate that upregulation of the transcription factors ELK4, SP1, and SP3 could account for the activation of LRG1 gene transcription observed in CRC. In addition, the levels of LRG1 in the plasma and urine have been identified as biomarkers for presurgical diagnosis in a broad range of malignancies.^[^ ^31^ ^]^ Given that the serum LRG1 protein level is increased in CRC, we propose that the serum LRG1 protein level could be a biomarker for treatment based on targeting the ELK4‐SP1/3 complex. In addition, a recent study reported that mechanical force induces LRG1 gene transcription mediated by ERK‐ELK1 during the progression of skin fibrosis.^[^ ^32^ ^]^ These findings imply that the TCF subfamily of transcription factors may generally regulate LRG1 gene transcription.

Given the prominent role of the ELK4‐SP1/3 complex in CRC tumorigenesis and progression, the ELK4‐SP1/3 complex might constitute a promising target for CRC treatment. Phosphorylation of TCF proteins by MAPKs enhances DNA binding of TCFs and affects the recruitment of coactivators/repressors by TCFs.^[^ ^21^ ^]^ In this study, we found that serum stimulates the activation of ERK, which leads to the phosphorylation of ELK4 and enhances the interactions between ELK4 and SP1/3. Thus, inhibition of MAPK activity could be a strategy for disrupting ELK4‐SP1/3 complex formation. Although MEK/ERK inhibitors have been demonstrated to improve the progression‐free survival of patients with BRAF mutant melanoma, they failed to show similar activity in other solid tumors.^[^ ^33^ ^]^ We propose that coinhibition of SP1/3 activity may enhance the inhibition of downstream target gene expression by disrupting ELK4‐SP1/3 complex formation. Mithramycin A (MMA), an FDA‐approved chemotherapeutic agent, exhibits antitumor properties by binding preferentially to GC‐rich sequences in DNA and competitively blocking the binding of SP1 to gene promoters. MMA has been used to treat myeloid leukemia and testicular carcinoma. Recently, MMA has also been demonstrated to have antitumor effects across a wide range of malignancies, including CRC,^[^ ^34^ ^]^ leading to a phase I/II clinical study of MMA monotherapy in thoracic malignancies (NCT02859415). Despite the potent antitumor effects, the application of MMA for cancer treatment is still limited due to its narrow therapeutic window and profound hepatotoxicity, which is probably due to the off‐targets of MMA other than SP1.^[^ ^35^, ^36^, ^37^ ^]^ Our findings indicate that combination treatment with MEK/ERK inhibitors and SP1 inhibitors could improve antitumor activity, constituting a new and promising strategy for CRC treatment. More studies and development of more specific SP1 inhibitors are warranted to validate the efficacy and safety of this therapeutic strategy targeting the ELK4‐SP1/3 complex in CRC.

## 4 Conclusion 

In conclusion, we describe a new mechanism by which ELK4 promotes tumorigenesis and tumor progression in CRC (Figure 7J). ELK4 cooperates with SP1 and SP3 instead of SRF to transcriptionally regulate LRG1, among others, to promote tumor angiogenesis, tumor growth, and metastasis. In particular, serum stimulation induces the phosphorylation of ELK4, which facilitates its interaction with SP1 and SP3. Targeting the ELK4‐SP1/3 transcriptional complex with combinations of MEK/ERK inhibitors and SP1 inhibitors is a promising strategy for CRC treatment.

## 5 Experimental Section

### Patient Samples

All human samples were collected in the Department of Colorectal Surgery, Xinhua Hospital, Shanghai Jiao Tong University School of Medicine, between January 2013 and December 2020. The study was approved by the ethics committee of Xinhua Hospital in 2013. Institutional review board approval and informed consent were obtained for all sample collections. The collection of clinicopathological data from the enrolled patients was approved by the ethics committee of Xinhua Hospital (XHEC‐NSFC‐2021‐326). Twenty‐four paired fresh CRC and normal tissue samples were collected for western blot and qPCR analyses to analyze the expression of ELK4. A total of 190 paired CRC and normal mucosa specimens were used to prepare tissue arrays for immunohistochemical (IHC) analysis of ELK4, SP1, SP3, CD31, and LRG1.

### Immunohistochemical Staining

Immunohistochemical staining was performed on formalin‐fixed, paraffin‐embedded sections according to a standard protocol. Details of the primary antibodies used in this study are presented in Table S3 (Supporting Information). Immunohistochemical staining in tumor and normal tissues was scored according to the following standards: the staining intensity was classified as 0 (lack of staining), 1 (mild staining), 2 (moderate staining), or 3 (strong staining), and the percentage of positive staining was designated 1 (< 25%), 2 (25–50%), 3 (51–75%), or 4 (> 75%). For each section, the semiquantitative score was calculated by multiplying these two values (resulting in a total score ranging from 0–12). Two histopathologists were assigned to review the slides and score the staining in a blinded manner.

### Cell Lines, Cell Culture, and Transfection

HEK293T, HCT116, LoVo, NCM460, CCD841, DLD1, HT29, RKO, SW480, and SW620 cells were purchased from the American Type Culture Collection (ATCC) and were authenticated by short tandem repeat analysis. All cells were cultured in DMEM/high glucose (HyClone) supplemented with 10% fetal bovine serum (BI), 100 units mL^−1^ penicillin, and 100 µg mL^−1^ streptomycin (Sangon Biotech) at 37 °C in 5% CO_2_. Cell transfection was performed using PEI (Polysciences) or Lipofectamine 2000 (Invitrogen) according to the manufacturers’ protocols. Lipofectamine RNAiMAX (Invitrogen) was used for siRNA transfection. The siRNA sequences are listed in Table S4 (Supporting Information).

### Generation of Stable Cells

ELK4 knockdown was accomplished with three independent shRNA oligonucleotides using a lentivirus‐mediated delivery system. The shRNA sequences are listed in Table S4 (Supporting Information). The shRNAs and pLKO.1 vector were cotransfected with psPAX2 and pMD2.G into HEK293T cells. After 48 h, the supernatant was collected, and the viral titer was measured before infection. HCT116 and LoVo cells were infected with lentivirus and selected with puromycin. The knockdown efficiency was determined by qPCR and western blot analyses. The full‐length human ELK4 sequence was cloned into the lentiviral expression vector pLVX.1‐puro to generate cells with stable ELK4 overexpression. Cells with stable LRG1 overexpression were generated by using the pQCXIH retroviral system.

### Plasmids

The full‐length ELK4 sequence was amplified from cDNA derived from HCT116 cells and was subsequently cloned into the pRK7‐FLAG vector; mutations were introduced by using a KOD Plus Mutagenesis Kit (TOYOBO) according to the manufacturer's protocol. The full‐length sequences and truncations of SP1 and SP3 were amplified by PCR and subcloned into the pcDNA‐HA expression vector.

### Coimmunoprecipitation and Western Blotting

After 24 h of transfection, the cells were harvested for lysis in lysis buffer (20 mm Tris‐HCl (pH 8.0), 150 mm NaCl, and 1% NP40) supplemented with protease inhibitor cocktails (Roche) for 30 min at 4 °C, followed by centrifugation. The supernatant was immunoprecipitated with the indicated agarose beads for 3 h at 4 °C. The beads were washed with lysis buffer three times, resuspended in loading buffer, and boiled at 95 °C for 10 min before loading onto SDS–PAGE gels. Immunoblotting was performed following standard procedures as previously described.^[^ ^10^ ^]^ The primary antibodies are listed in Table S3 (Supporting Information).

### RNA Isolation and qRT–PCR

Total RNA was extracted using TRIzol reagent (Invitrogen) and was then reverse‐transcribed into cDNA using a PrimeScript RT Kit (TaKaRa). Quantitative reverse transcription‐PCR was performed using a SYBR Green Kit (TaKaRa). The primer sequences are listed in Table S5 (Supporting Information).

### Cell Proliferation and Migration Assays

For the cell proliferation assay, stable cell lines were seeded in 96‐well plates at 1000 cells per well. The cell proliferation capability was measured by a Cell Counting Kit‐8 assay. The migration assay was performed using a 24‐well Transwell chamber system (Corning). Briefly, 1 × 10^5^ cells suspended in 100 µL of FBS‐free medium were placed in the upper compartment, and 600 µL of medium containing 10% FBS was added to the lower compartment. After incubation for 48 h, the cells were first fixed and then stained with crystal violet to evaluate the migration ability.

### Endothelial Cell Tube Formation Assay

Growth factor‐reduced Matrigel (BD Biosciences) was added to 96‐well plates and allowed to polymerize at 37 °C. HUVECs (5000 cells per well) were seeded in plates and cultured with the supernatant from stable cell lines. After incubation for 6–8 h, endothelial cells were photographed and quantitatively analyzed.

### Xenograft Tumor Models

The CDX and PDX models were established as previously described.^[^ ^38^ ^]^ After the tumor volume was approximately 100 mm^3^, mice were randomly assigned to the indicated groups and treated with vehicle, 0.1 mg kg^−1^ MMA (i.p., three times a week), 5 mg kg^−1^ U0126 (i.v., twice a week), or a combination of MMA and U0126 for 2 weeks. Tumor sizes were measured and monitored at the indicated time points, and tumor volumes were calculated with the following formula: 0.5 × (largest diameter) × (smallest diameter)^2^. At the experimental endpoint, the mice were sacrificed, and the tumors were harvested, imaged, and weighed. All procedures with mice were approved by the ethics committee of Xinhua Hospital affiliated with Shanghai Jiaotong University School of Medicine (XHEC‐NSFC‐2020‐115).

### Hepatic Metastasis and Lung Metastasis Models

For the colorectal cancer hepatic metastasis model, stable cell lines were surgically injected into the spleens via hemisplenectomy, as described previously.^[^ ^10^ ^]^ In the lung metastasis model, 1 × 10^6^ cells of the stable cell lines were resuspended in 100 µL of phosphate‐buffered saline (PBS) and injected into the lateral tail veins of 5‐week‐old nude mice. After 6 weeks, the mice were sacrificed, and the livers and lungs were collected and fixed with 4% paraformaldehyde solution, prior to H&E staining to analyze the metastatic nodules. The areas of liver and lung metastases were quantified by ImageJ software.

### AOM‐DSS Model of Colorectal Tumorigenesis

*Elk4* knockout mice on a C57BL genetic background were purchased from Cyagen Bioscience. Paired *Elk4^−/−^* and *WT* littermate control mice were subjected to model induction at the age of 8 weeks and were intraperitoneally injected with AOM (10 mg kg^−1^, dissolved in PBS; Sigma) on Day 1. Beginning on Day 2, the mice were administered three cycles of DSS (MP Biologicals) dissolved in the drinking water at stepwise increasing concentrations of 1.25%, 1.5%, and 1.75%. During every cycle, the mice drank DSS‐containing water for 7 consecutive days and were then provided regular drinking water for 14 days for recovery. Mice were sacrificed on Day 65, and colons were collected and cut open longitudinally to determine the tumor numbers and sizes.

### Intestinal Organoid Culture and Organoid Viability Assay

Intestinal organoids from *WT* and *Elk4^−/−^* mice were established and cultured in droplets of Matrigel (Thermo Fisher), and the medium was exchanged every 48 h. The obtained colonic organoids were cultured in Advanced DMEM/F12 (Thermo Fisher) containing 10% HEPES (Thermo Fisher), 1 × GlutaMAX (Thermo Fisher), 1 × B27 (Thermo Fisher), murine EGF (50 ng mL^−1^, Sigma), murine Noggin (100 ng mL^−1^, PeproTech), 0.5 mm A83‐01 (Tocris), 3 µm SB202190 (Sigma), 1 µM nicotinamide (Sigma), 50% Wnt3A‐conditioned medium and 20% R‐spondin‐conditioned medium. Patient‐derived CRC organoids were established and cultured in droplets of Matrigel (Thermo Fisher), and the medium was exchanged every 48 h. Detailed procedures were described previously.^[^ ^38^ ^]^ Seventy‐two hours after seeding, organoids were treated with the indicated concentration of U0126 (Selleck) or mithramycin A (MCE) for 48 h. The viability of organoids was evaluated by a CellTiter‐Glo luminescent cell viability assay (Promega).

### Immunoprecipitation‐Mass Spectrometry (IP‐MS) Analysis

HEK293T cells were seeded in 60 cm plates and transfected with Flag‐ELK4 or empty vector for 48 h. Cells were harvested and subjected to co‐IP with an anti‐Flag antibody as mentioned above. The immunoprecipitated samples were separated by SDS–PAGE, and the gels were stained with Coomassie Blue. The immunoprecipitated protein fractions were subjected to GC–MS analysis in a GC–MSD system (Agilent Technologies). The levels of serine and glycine were normalized to the cell counts.

### RNA‐Seq Analysis

Total RNA was extracted from the indicated HCT116 cells using TRIzol (Invitrogen) as mentioned above. RNA was treated with DNase I (Qiagen) to remove any contaminating genomic DNA, and poly‐(T) oligo‐attached magnetic beads (Invitrogen) were then used for purification. The purified RNAs were subjected to fragmentation and library construction and were then sequenced on the Illumina HiSeqTM 2500 platform. Expression levels, represented as fragments per kilobase of transcript per million mapped reads (FPKM) values, were calculated by Cufflinks and normalized to the total number of mapped reads per sample. Differentially expressed genes (DEGs) were identified as those with a fold change (FC) ≥ 2 and FDR < 0.05.

### Gene Set Enrichment Analysis (GSEA)

GSEA was carried out with GSEA software (version 4.1.0) and the Molecular Signatures Database (MSigDB).^[^ ^39^ ^]^ The GSEA preranked method, in which the differentially expressed genes identified by RNA‐seq analysis were ranked by log_2_(FC) values, was used to conduct the GSEA.

### ChIP‐qPCR and ChIP‐Seq Analysis

HCT116 cells were transfected with Flag‐ELK4 for 48 h and were then harvested for chromatin immunoprecipitation (ChIP) with a Magna ChIP Kit (Merck, 17–610). Briefly, cells were crosslinked with 1% formaldehyde for 10 min at room temperature, and 1.25 m glycine was then added for 5 min at room temperature to terminate crosslinking. After washing, lysis, and sonication, the chromatin fraction was incubated with an anti‐Flag antibody (CST, 14 793) or control anti‐IgG (CST, 3900) overnight at 4 °C. Chromatin‐bound beads were subjected to extensive washing and elution. Eluted chromatin was decrosslinked for 2 h at 65 °C and was then treated with proteinase K for protein digestion. The obtained DNA samples were further purified and prepared for subsequent qPCR analysis, multiplexed library preparation, and deep sequencing. The qPCR primers were included in Table S5 (Supporting Information).After quality control, the clean reads were aligned to the human reference genome (GRCh38) with Bowtie 2.^[^ ^40^ ^]^ Peak calling was performed using default parameters in MACS2 with input as the negative control.^[^ ^41^ ^]^ DeepTools2 was used to generate heatmaps and perform genome‐wide correlation analyses.^[^ ^42^ ^]^ Mapped reads were visualized using Integrated Genomics Viewer (IGV).^[^ ^43^ ^]^

### Proximity Ligation Assay

A proximity ligation assay (PLA) was performed using a Duolink In Situ Detection Kit (Sigma) according to the manufacturer's protocol. Briefly, CRC cells were fixed with 4% PFA for 30 min, permeabilized with 0.5% Triton X‐100 for 10 min, and blocked with blocking solution for 1 h. After blocking, cells were incubated with primary antibodies against ELK4 (1:100, Abnova, H00002005‐B01P) and SP1 (1:100, ABclonal, A19649) or SP3 (1:100, Abcam, ab227856) for 1 h at room temperature prior to incubation with Duolink PLA probes for 1 h in a humidified chamber at 37 °C. Samples were incubated first with Ligation Solution for 30 min and then with Amplification Solution for 100 min in a dark, humidified chamber at 37 °C. Finally, coverslips were air‐dried and mounted with Mounting Medium with DAPI (Sigma). After mounting, the cells were photographed, and the number of nuclear foci per cell was quantified using ImageJ software.

### Luciferase Reporter Assay

The LRG1 promoter (−4500/+100 bp) and enhancer (*1/*1500 bp) were cloned into the pGL3‐Basic reporter vector. HCT116 cells were seeded in 24‐well plates in triplicate and cotransfected with the reporter plasmid and Renilla luciferase plasmid together with other plasmids as indicated for 48 h. Luciferase activity was measured using a Dual‐Luciferase Reporter Assay System (Promega) according to the manufacturer's protocol, and reporter gene activity was determined by normalizing firefly luciferase activity to Renilla luciferase activity.

### Construction and Testing of the ESGS‐Related Prognostic Model for CRC Patients

The mRNA expression data, as well as the clinical OS events and times, were downloaded from the TCGA‐COAD dataset and GEO datasets (GSE29621 and GSE38832). Patients without complete survival information were excluded from further analysis. The TCGA‐COAD dataset was used as the training set (*n* = 447), whereas the GSE29621 and GSE38832 datasets were merged into an independent test set (*n* = 157). First, univariate Cox regression analysis was performed to screen the ELK4‐SP1/3 complex‐regulated gene set (ESGS) related to OS, and *P* < 0.05 was considered significant. Subsequently, LASSO regression was conducted to prevent overfitting of the models. Finally, stepwise multivariate Cox regression analysis was carried out to evaluate the risk signatures of the predictive model and obtain their regression coefficients. The risk score for each patient was calculated with the following formula:

(1) $$\mathrm{Risk}\;\mathrm{score}=\sum_{i\;=\;1}^{n}\left(Coef_{i}\times Expr_{i}\right)$$

The patients were divided into a high‐risk and a low‐risk group based on the median risk score as the cutoff value. After that, survival analysis and ROC analysis were conducted to evaluate the predictive ability of the ESGS‐based signature for OS.

### Statistical Analysis

All statistical analyses were performed with GraphPad Prism 7.0 and SPSS 22.0. Data are presented as the mean ± S.D. values unless otherwise noted in the figure legends. A paired or unpaired two‐tailed Student's *t* test was used to determine the statistical significance of differences between two groups. One‐way ANOVA was used to assess the statistical significance for the experiments with >2 independent groups. For the CCK8 and xenograft growth curve assays, two‐way ANOVA was performed to assess the statistical significance. Relationships between ELK4 expression and clinicopathologic factors were analyzed by the chi‐square test. Survival analyses were performed using the Kaplan–Meier method, and significance was assessed by the log‐rank test. Pearson and Spearman correlation analyses were used to determine correlations between mRNA expression levels and protein expression levels, respectively. A *P* value < 0.05 was considered statistically significant (*, *P* < 0.05; **, *P* < 0.01; ***, *P* < 0.001).

## Conflict of Interest

The authors declare no conflict of interest.

## Ethics Approval Statement

This study was approved by the ethics committee of Xinhua Hospital affiliated with Shanghai Jiaotong University School of Medicine (XHEC‐NSFC‐2020‐115 and XHEC‐NSFC‐2021‐326).

## Author Contributions

Z.Z., Y.G., Y.L., and R.D. contributed equally to this work. Z.Z., Y.G., and C.‐Y.L. conceived and designed the study. Z.Z, Y.G, R.D., and Z.H. performed the experiments. Z.Z. and Y.L. analyzed and interpreted the data. Y.L., L.C., and P.D. provided patient samples and performed related analyses. Z.Z., W.Y., and C.‐Y.L. wrote and revised the manuscript. A.G. and C.‐Y.L. supervised the study. All authors have approved the manuscript for submission and consented for publication.

## Supporting information

Supporting Information

## Data Availability

The data that support the findings of this study are openly available in GEO at https://www.ncbi.nlm.nih.gov/geo/query/acc.cgi?acc=GSE179294, reference number 179294.
