# Supplementary material for: ELK4 Promotes Colorectal Cancer Progression by Activating the Neoangiogenic Factor LRG1 in a Noncanonical SP1/3‐Dependent Manner
Source: Adv Sci (Weinh). 2023 Oct 2;10(32):2303378. doi: 10.1002/advs.202303378 (PMC10646254; doi:10.1002/advs.202303378)
Supplement: Supplementary file 1 — Supporting Information [file ADVS-10-2303378-s001.pdf]

ADVANCED SCIENCE
Open Access

Supporting Information

for *Adv. Sci.*, DOI 10.1002/advs.202303378

ELK4 Promotes Colorectal Cancer Progression by Activating the Neoangiogenic Factor LRG1 in a Noncanonical SP1/3-Dependent Manner

Zhehui Zhu, Yuegui Guo, Yun Liu, Rui Ding, Zhenyu Huang, Wei Yu, Long Cui, Peng Du, Ajay Goel* and Chen-Ying Liu**

Supporting Information

ELK4 Promotes Colorectal Cancer Progression by Activating the Neoangiogenic Factor LRG1 in a Noncanonical SP1/3-dependent Manner

Zhehui Zhu, Yuegui Guo, Yun Liu, Rui Ding, Zhenyu Huang, Wei Yu, Long Cui, Peng Du [*] *Ajay Goel,* [*] *Chen-Ying Liu*[*]

Supplementary figures and figure legends

Sup. Fig. S1

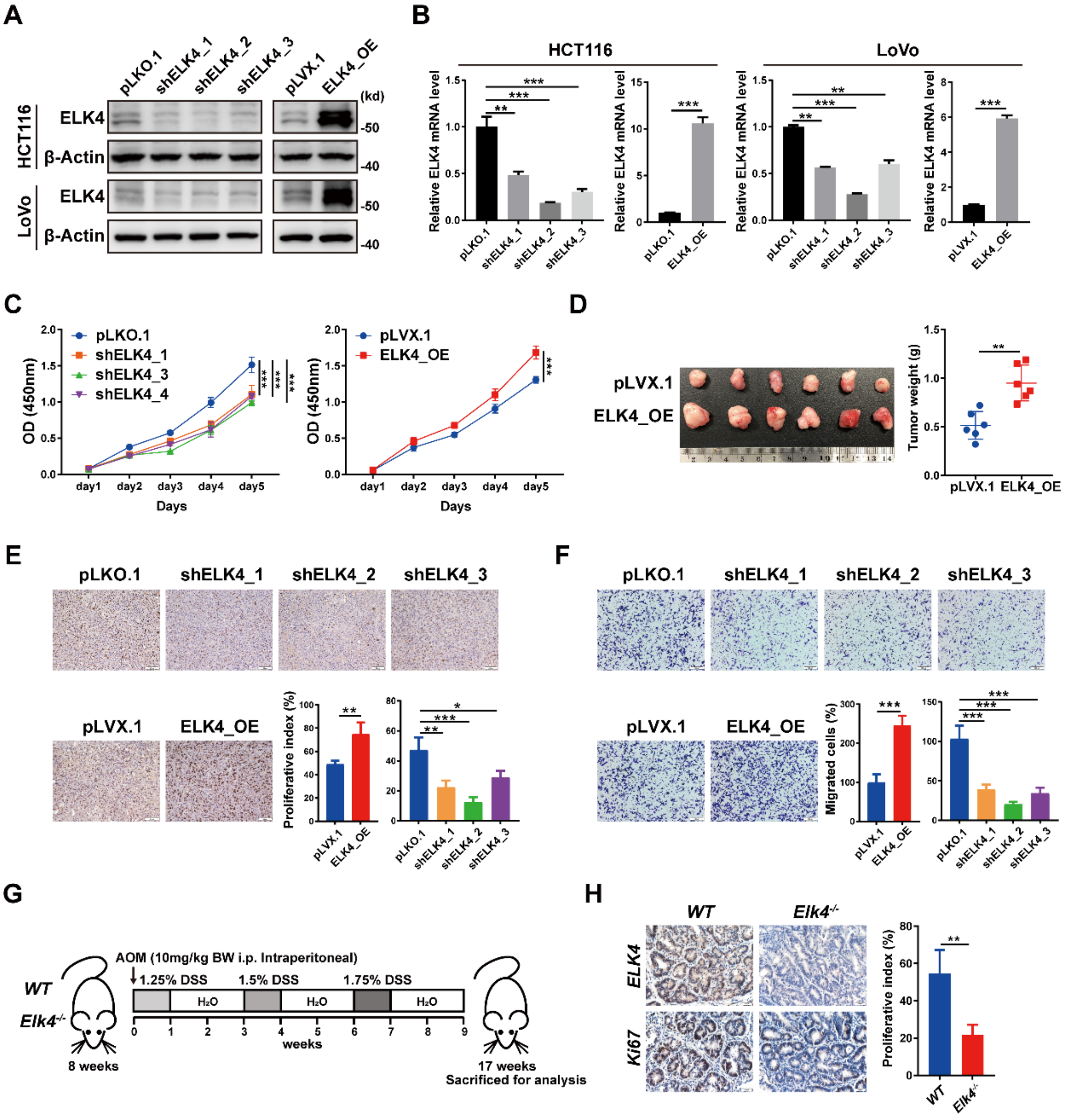

Supplementary Figure 1. Related to Figure 1.

(A and B) Generation of HCT116 and LoVo cells with ELK4 knockdown or overexpression. Western blot (A) and qPCR (B) analyses were performed to detect the expression of ELK4 in these cells.

(C) CCK8 assays of the proliferation of LoVo cells with ELK4 knockdown or overexpression.

(D) Representative images and statistical analysis of ELK4-overexpressing HCT116 xenograft tumors (n=6).

(E) Immunohistochemical analysis of Ki67 in xenografts derived from HCT116 cells

with ELK4 knockdown or overexpression.

(F) Transwell assays to evaluate the migration ability of LoVo cells with ELK4 knockdown or overexpression.

(G) Schematic illustration of the procedure for chemical induction of colorectal cancer in mice via intraperitoneal injection of AOM and three subsequent cycles of DSS administration in drinking water.

(H) Representative images and quantification of ELK4 and Ki67 staining in colon tumors form *WT* and *Elk4*$^{-/-}$ mice after AOM/DSS model establishment.

One-way ANOVA (B, E and F), Student's t test (B, D, E, F and H) and Two-way ANOVA (C) were performed to assess the statistical significance. The data are presented as the mean ± S.D. values. * $P < 0.05$, ** $P < 0.01$, *** $P < 0.001$.

Sup. Fig. S2

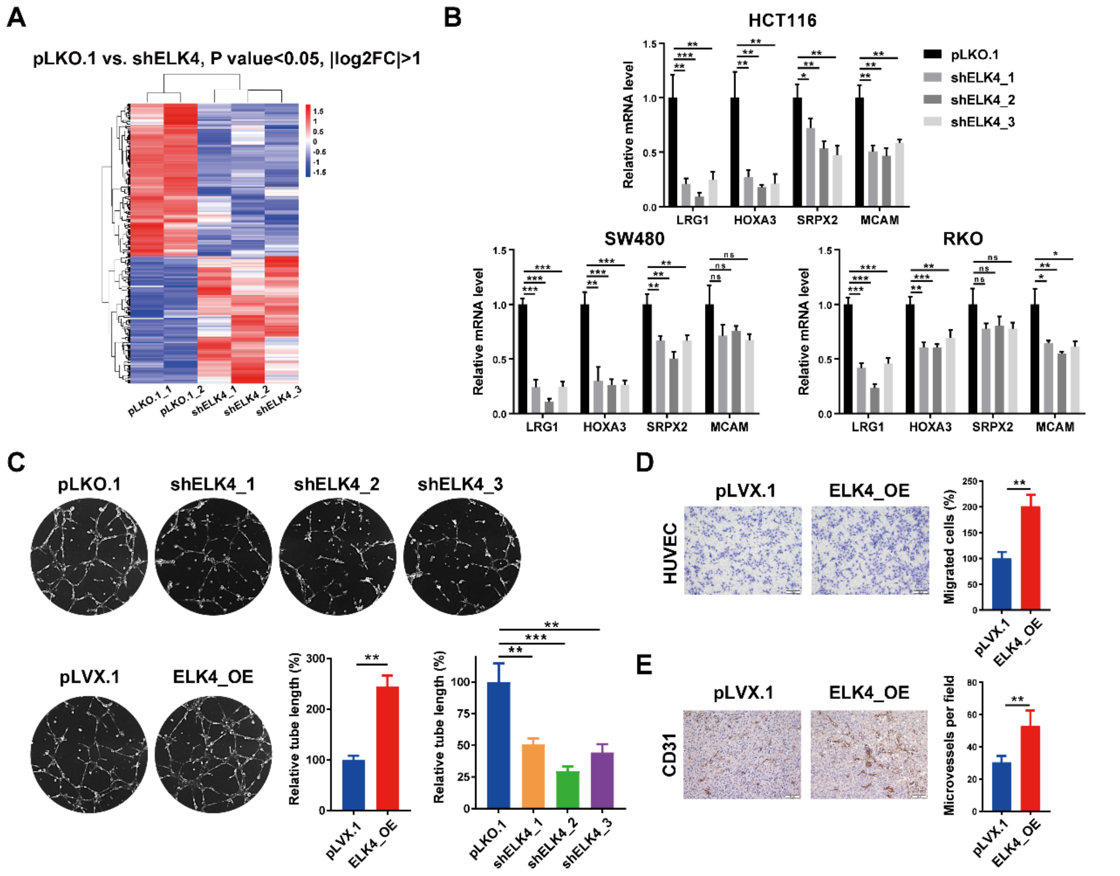

Supplementary Figure 2. Related to Figure 2.

(A) Heatmap of significantly differentially expressed genes (FC > 2.0, P < 0.05) between pLKO.1- and shELK4-transduced HCT116 cells identified by RNA-seq.

(B) The downregulated genes in shELK4 CRC cells were confirmed by qPCR analysis.

(C) Representative images and statistical analysis of HUVEC tube formation of in the presence of CM from pLKO.1-, shELK4-, pLVX.1- or ELK4_OE vector-transduced LoVo cells.

(D) Representative images and statistical analysis of Transwell assays of HUVECs in the presence of CM from pLVX.1- or ELK4_OE vector-transduced HCT116 cells.

(E) Representative images and statistical analysis of IHC staining for CD31 in xenografts derived from pLVX.1- and ELK4_OE vector-transduced HCT116 cells.

One-way ANOVA (B and C) and Student’s t test (C, D and E) were performed to assess the statistical significance. The data are presented as the mean ± S.D. values. ** P < 0.01, *** P < 0.001.

Sup. Fig. S3

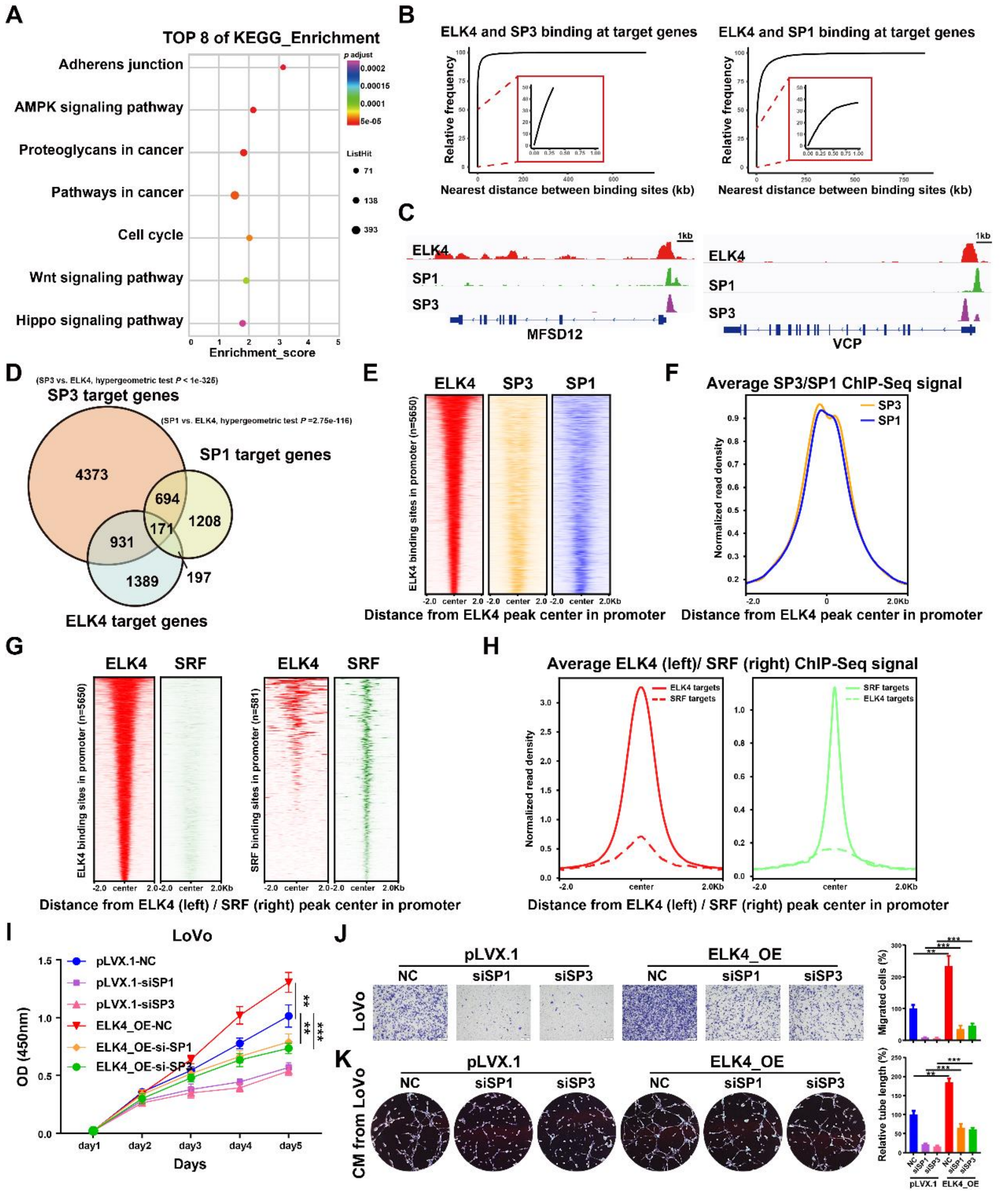

Supplementary Figure 3. Related to Figure 3.

(A) KEGG enrichment analysis of the overlapping target genes bound by ELK4, SP1 and SP3.

(B) Cumulative frequency distribution of the smallest distance between ELK4 and SP3 (left) or SP1 (right) binding sites associated with each shared target gene. Distances were calculated by using the peak coordinates of each factor nearest one another.

(C) Genome Browser representation of ELK4 (red), SP1 (green) and SP3 (purple) peaks

in MFSD12 and VCP genes.

(D) Venn diagram displaying the overlap between ELK4, SP1 and SP3 target genes with ELK4/SP1/SP3 binding to promoter in HCT116 cells.

(E) Heatmaps of ChIP-seq data for ELK4, SP1 and SP3 in HCT116 cells. All peaks in each heatmap are centered ± 2.0 kb from the ELK4 peaks in ELK4-bound promoters.

(F) Normalized read density (per bp per peak) for ELK4, SP1 and SP3 plotted in the region ± 2.0 kb from the ELK4-bound promoters.

(G) Heatmaps of ChIP-seq data for ELK4 and SRF peaks in the ELK4 (left) and SRF (right) targeting promoters.

(H) Normalized read density (per bp per peak) for ELK4 and SRF plotted in the region ± 2.0 kb from the ELK4-bound (left) or SRF-bound (right) promoters.

(I and J) ELK4-overexpressing LoVo cells were transfected with control, SP1 and SP3 siRNAs for 2 days and then analyzed by CCK8 (I) and Transwell assays (J).

(K) Representative images and statistical analysis of HUVEC tube formation incubated with CM from ELK4-overexpressing LoVo cells transfected with control, SP1 and SP3 siRNAs.

Two-way ANOVA (I) and one-way ANOVA (J and K) were performed to assess the statistical significance. The data are presented as the mean ± S.D. values. ** $P < 0.01$, *** $P < 0.001$.

Sup. Fig. S4

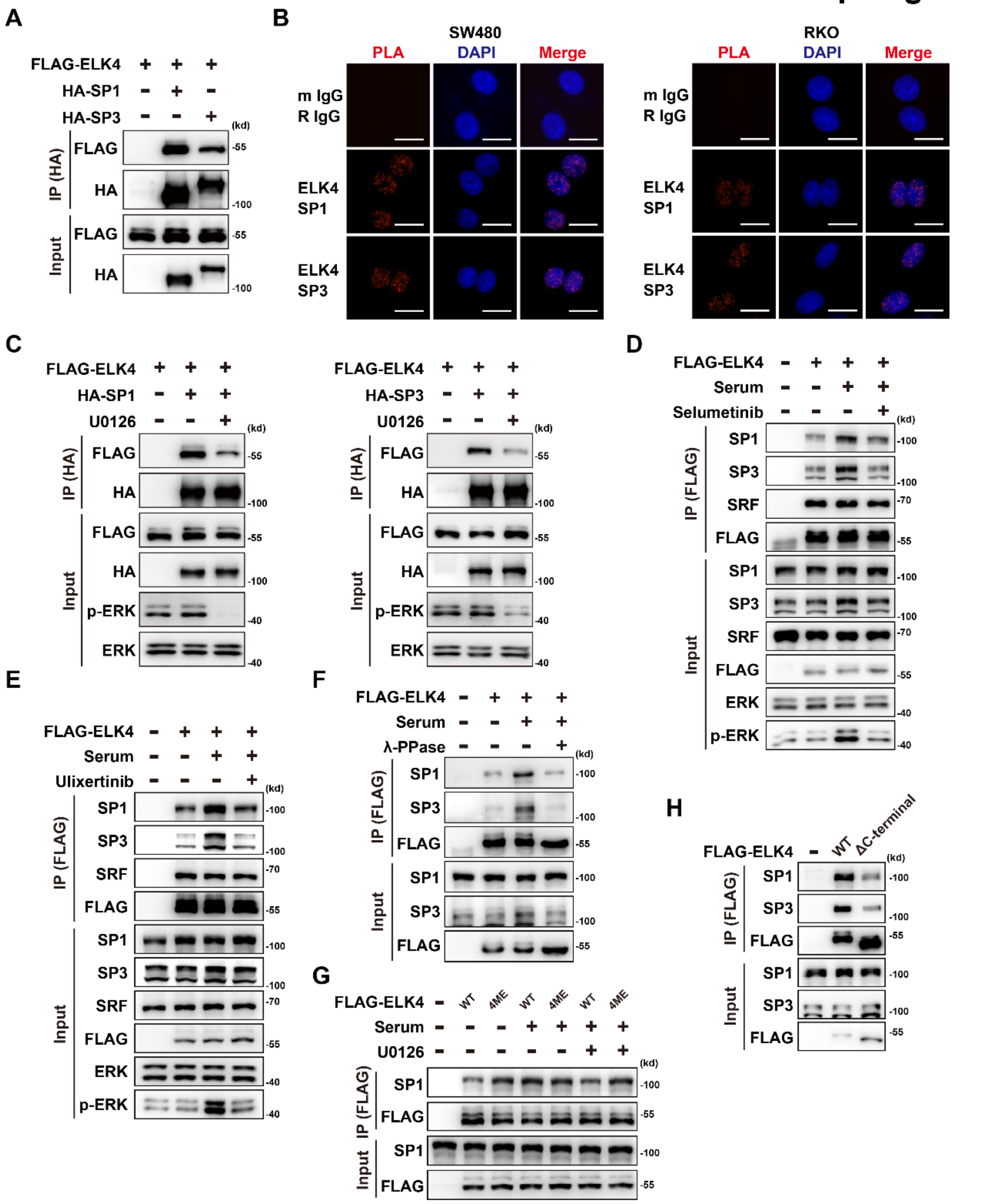

Supplementary Figure 4. Related to Figure 4.

(A) Coimmunoprecipitation (Co-IP) to evaluate the interactions between FLAG-ELK4 and HA-SP1 and HA-SP3 in HCT116 cells.

(B) Proximity ligation assay (PLA) to evaluate the interactions of ELK4 with SP1 and SP3 in SW480 and RKO cells. The red dots indicate protein interactions (scale bars:10 μm).

(C) U0126 suppressed the serum-induced interactions between ELK4 and SP1 and SP3

in HCT116 cells. HCT116 cells were transfected with the indicated plasmids for 24 h and were then cultured in the presence or absence of U0126 (10 μM) for 12 h before co-IP assay.

(D, E) Selumetinib (D) and Ulixertinib (E) suppressed the serum-induced interactions between ELK4 and SP1 and SP3 in HCT116 cells. HCT116 cells were transfected with the indicated plasmids for 24 h and were then cultured in the presence or absence of Selumetinib (D) and Ulixertinib (E) for 12 h before co-IP assay.

(F) λ-protein phosphatase (λ-PPase) decreased the interaction between ELK4 and SP1 /SP3. HCT116 cells were transfected with the indicated plasmids for 24 h and the cell lysates were treated with λ-PPase for 1h and then subjected to immunopreciation.

(G) HCT116 cells were transfected with the indicated plasmids for 24 h and were then cultured in the presence or absence of U0126 (10 μM) for 12 h.

(H) HCT116 cells were transfected with ELK4 wild-type and C-terminal deletion plasmids for 24 h and then subjected to co-IP assay.

Sup. Fig. S5

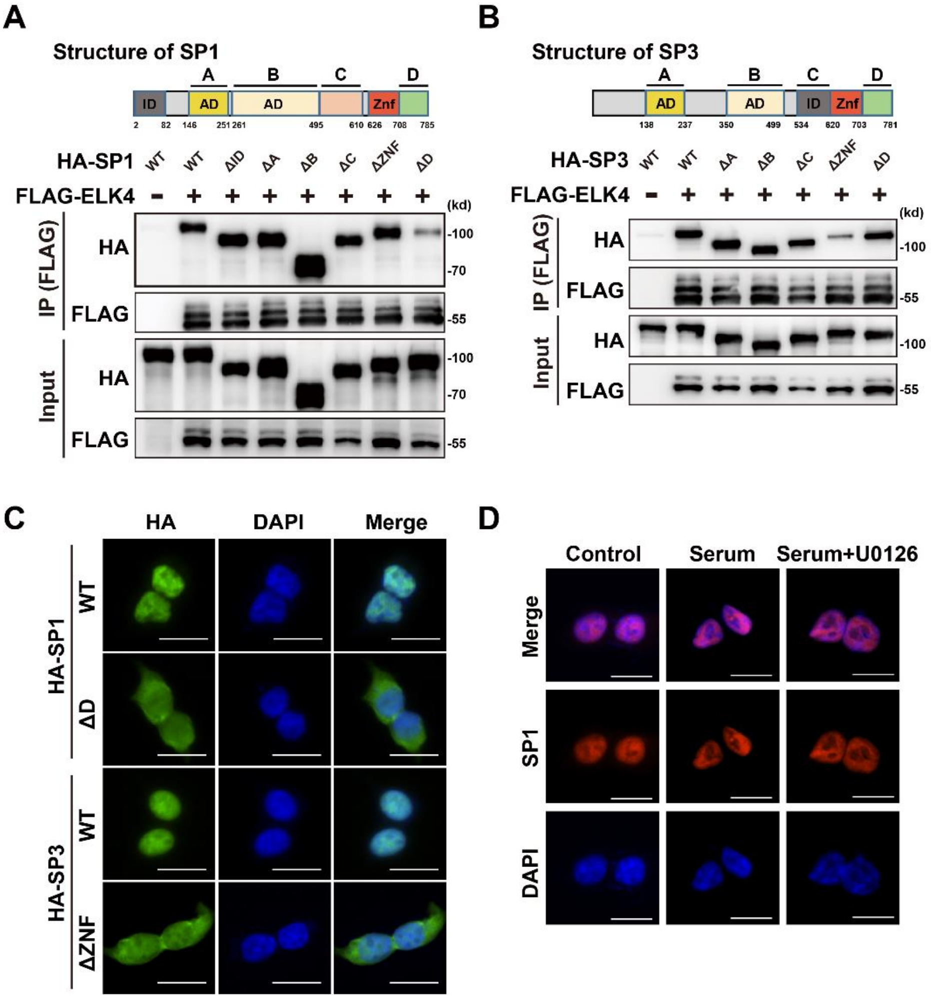

Supplementary Figure 5. Related to Figure 4.

(A and B) The domain organization maps of the full-length SP1 (A) and SP3 (B) proteins are shown schematically. Co-IP analysis was performed to map the domains participating in the interactions of the HA-SP1 and HA-SP3 constructs with Flag-ELK4.

(C) Immunofluorescence assay of the full-length SP1/3 and truncated SP1/3 in HCT116 cells.

(D) Immunofluorescence assay of the endogenous SP1 in serum-starved HCT116 cells treated serum or serum plus U0126 treatment.

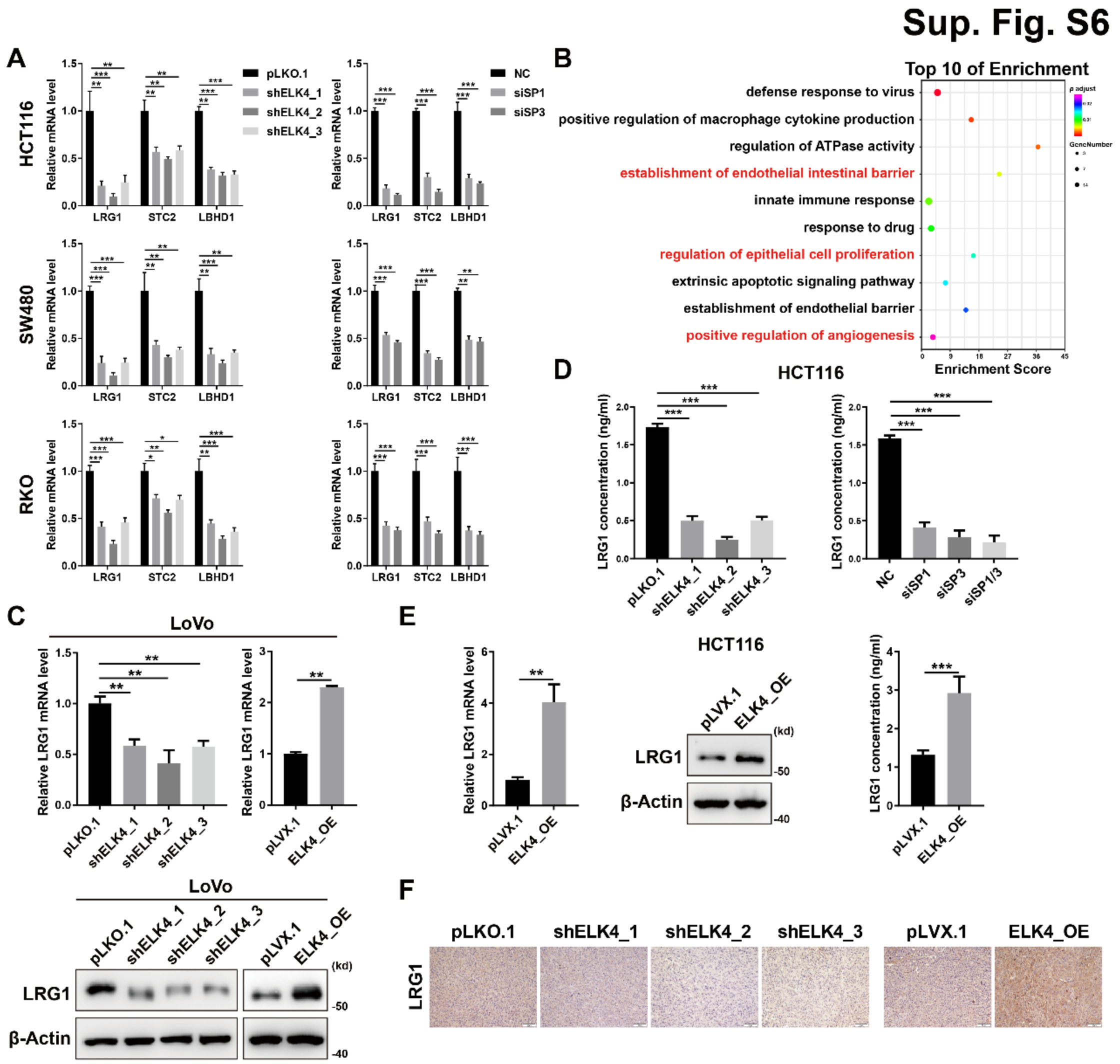

Supplementary Figure 6. Related to Figure 5.

(A) qPCR analysis of the mRNA levels of LRG1, STC3 and LBHD1 in HCT116 cells with ELK4 or SP1/3 knockdown.

(B) GO analysis based on the newly identified ELK4-SP1/3 co-regulated genes.

(C) qPCR (up) and western blot (down) analysis of the LRG1 mRNA and protein level in LoVo cells with ELK4 knockdown or overexpression.

(D) ELISA analysis of the LRG1 protein level in the condition medium from HCT116 cells with ELK4 or SP1/3 knockdown.

(E) qPCR, western blot analysis of the LRG1 mRNA and protein level in HCT116 cells with ELK4 overexpression. ELISA analysis of the LRG1 protein level in the condition medium from HCT116 cells with ELK4 overexpression.

(F) Immunohistochemical staining indicated the expression of LRG1 in xenografts

derived from ELK4 knockdown or ELK4-overexpressing HCT116 cells (scale bars = 50 μm).

One-way ANOVA (A, C and D) and Student's t test (C and E) were performed to assess the statistical significance. The data are presented as the mean ± S.D. values. ** $P < 0.01$, *** $P < 0.001$.

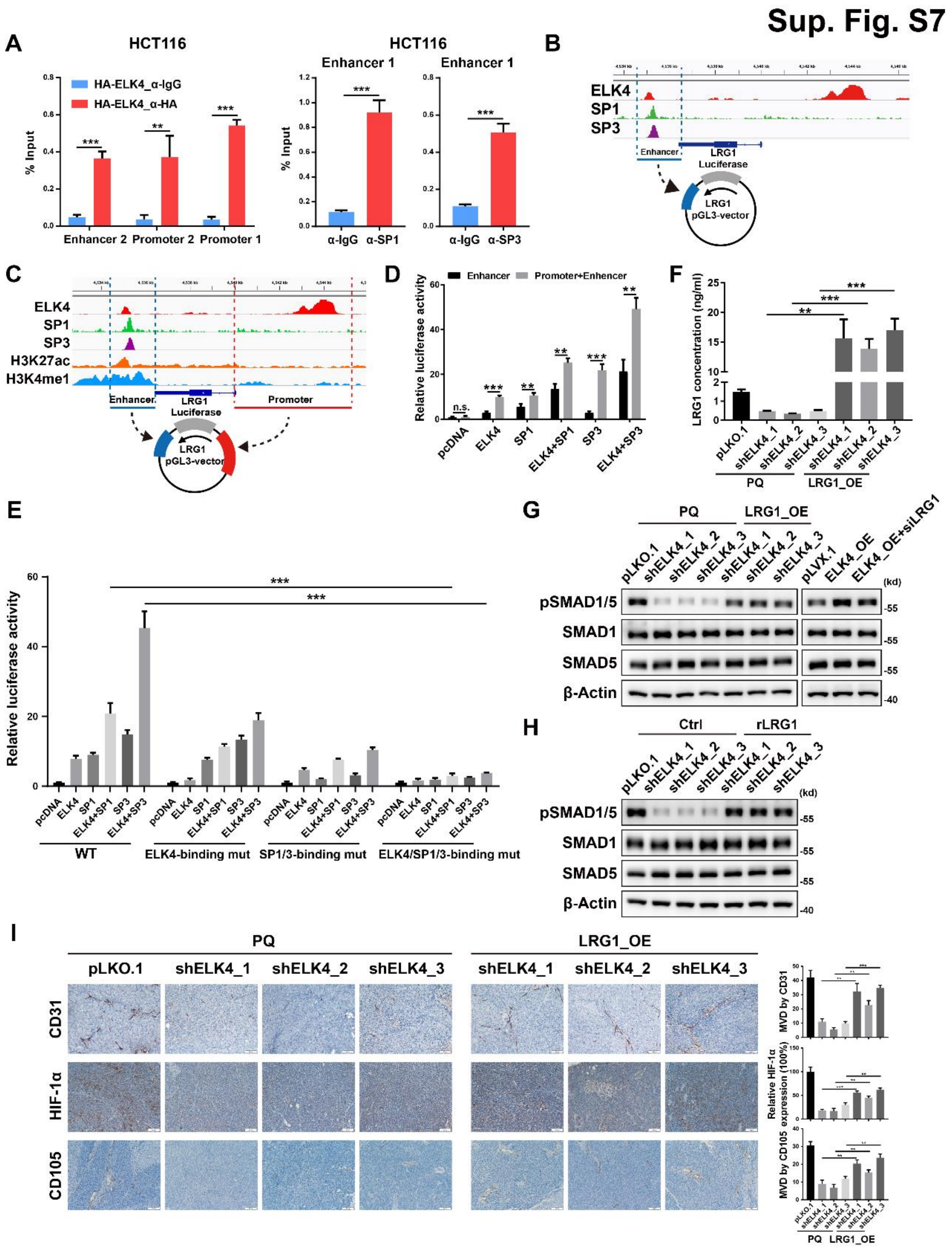

Supplementary Figure 7. Related to Figure 5.

(A) ChIP-qPCR analysis of ELK4 and SP1/3 binding to the LRG1 gene locus.

(B) Schematic depiction of the LRG1 luciferase reporter construct containing the LRG1 enhancer, as identified by the ChIP-seq data for ELK4, SP1 and SP3.

(C) Schematic depiction of the LRG1 luciferase reporter construct containing both the LRG1 enhancer and promoter, as identified by the ChIP-seq data for ELK4, SP1 and

SP3, H3K27ac and H3K4me1.

(D) Luciferase assay of HCT116 cells cotransfected with the LRG1 reporter containing the enhancer (with or without the promoter) and the empty vector or the indicated plasmids of ELK4, SP1 and SP3.

(E) Luciferase assay of HCT116 cells with the LRG1 reporter (promoter+enhancer) with or without mutation of ELK4 binding sites or SP1/3 binding sites.

(F) ELISA analysis of the LRG1 protein level in the condition medium from ELK4 knockdown HCT116 cells overexpressing LRG1.

(G) Western blot analysis of the p-SMAD1/5, SMAD1 and SMAD5 in HUVEC treated with the CM from ELK4 knockdown HCT116 cells or with rescued LRG1 expression (left) and ELK4 overexpression HCT116 cells or with LRG1 knockdown (right).

(H) Western blot analysis of the p-SMAD1/5, SMAD1 and SMAD5 in HUVEC treated with the CM from ELK4 knockdown HCT116 cells with 500 ng/ml rLRG1.

(I) Immunohistochemical staining indicated the expression of CD31, HIF-1α and CD105 in xenografts derived from ELK4 knockdown HCT116 cells overexpressing LRG1 (scale bars = 50 μm).

Student's t test (A and D) and One-way ANOVA (E, F and I) were performed to assess the statistical significance. The data are presented as the mean ± S.D. values. * $P < 0.05$, ** $P < 0.01$, *** $P < 0.001$.

Sup. Fig. S8

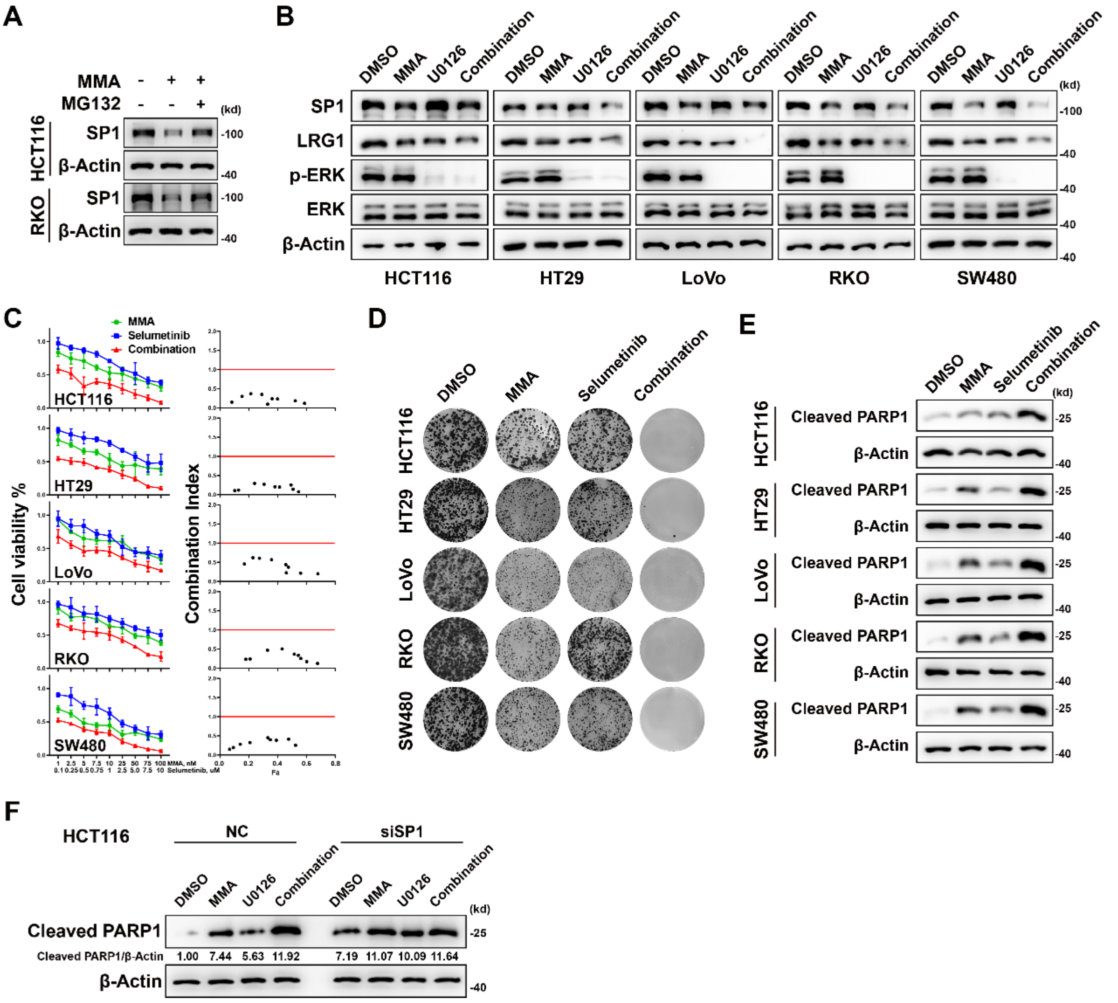

Supplementary Figure 8. Related to Figure 6.

(A) Western blot analysis of SP1 protein in HCT116 and RKO cells treated with MMA or MMA+MG132.

(B) Western blot analysis of indicated protein levels in HCT116, HT29, LoVo, RKO and SW480 cells treated with vehicle, 50 nM MMA, 5 μM U0126 or the combination of MMA and U0126.

(C) Cell proliferation was assessed following 48 h of exposure to the indicated concentrations of MMA, Selumetinib or the combination in HCT116, HT29, LoVo, RKO and SW480 cells. CI values for the various combinations were calculated using CompuSyn. A CI < 1.0 indicates a synergistic effect.

(D) Colony formation assays showed a synergistic response to the combination of Selumetinib and MMA in HCT116, HT29, LoVo, RKO and SW480 cells

(E) Representative western bolts of cleaved PARP1 in HCT116, HT29, LoVo, RKO and SW480 cells treated with vehicle, 50 nM MMA, 100 nM Selumetinib or the combination of MMA and Selumetinib.

(F) Representative western bolts of cleaved PARP1 in control and SP1 knockdown HCT116 cells treated with vehicle, 50 nM MMA, 5 μM U0126 or the combination of MMA and U0126.

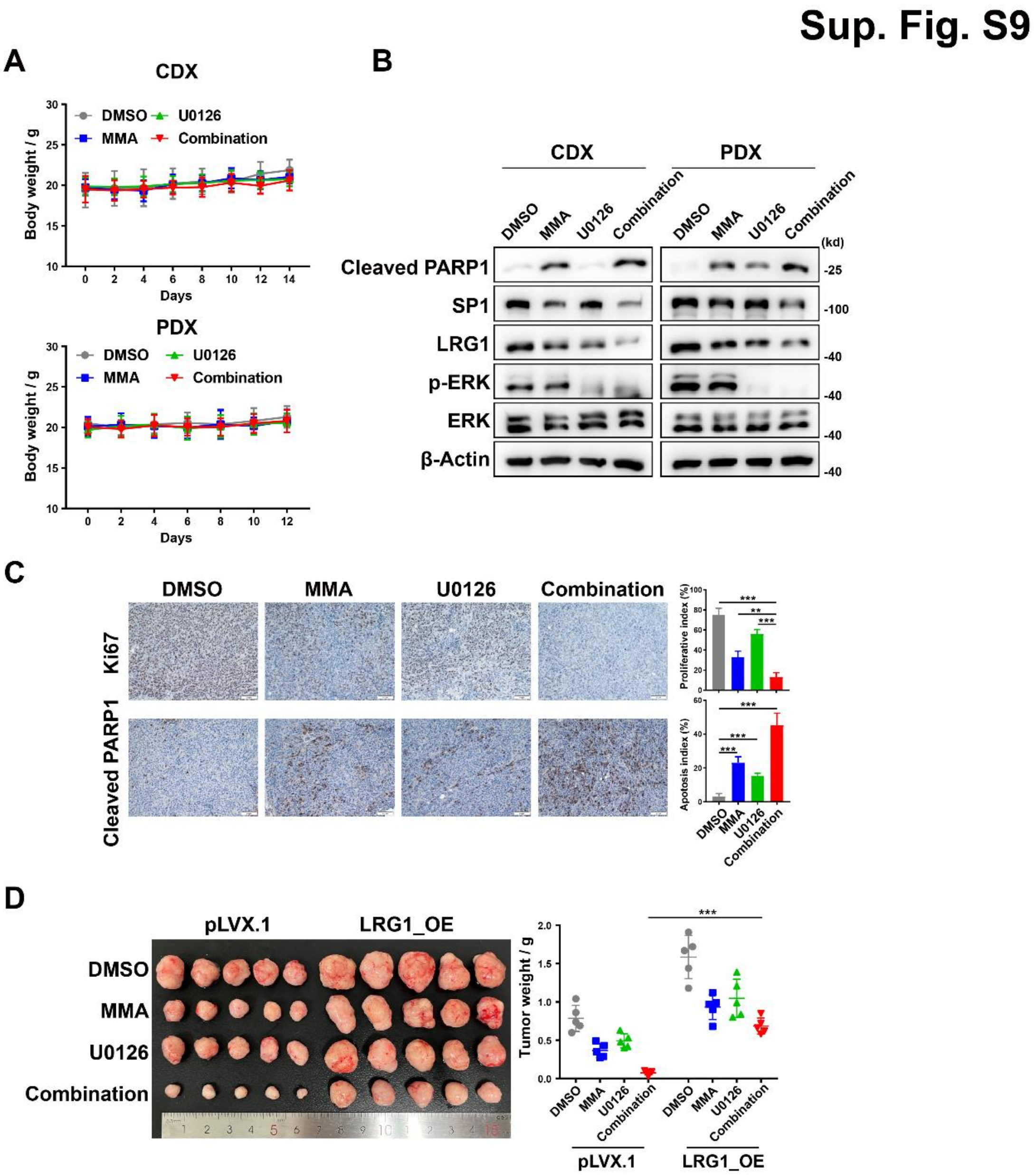

Supplementary Figure 9. Related to Figure 6.

(A) The body weight of nude mice treated with vehicle, 0.5 mg/kg MMA, 10 mg/kg U0126 or the combination of MMA and U0126 (n = 5 mice per group).

(B) Representative western blot analysis of indicated protein levels in HCT116 cell-derived and CRC patient-derived xenografts harvested from nude mice treated with vehicle, 0.5 mg/kg MMA, 10 mg/kg U0126 or the combination of MMA and U0126.

(C) Immunohistochemical staining indicated the expression of Ki67 and cleaved PARP1 in CDXs treated with vehicle, 0.5 mg/kg MMA, 10 mg/kg U0126 or the combination of MMA and U0126 (scale bars = 50 μm).

(D) Representative images of control and LRG1 overexpression HCT116 cell-derived

xenografts harvested from nude mice treated with vehicle, 0.5 mg/kg MMA, 10 mg/kg U0126 or the combination of MMA and U0126 (n = 5 mice per group). Xenograft weight were measured for statistical analysis.

One-way ANOVA (C, D) were performed to assess the statistical significance. The data are presented as the mean ± S.D. values. * $P < 0.05$, ** $P < 0.01$, *** $P < 0.001$.

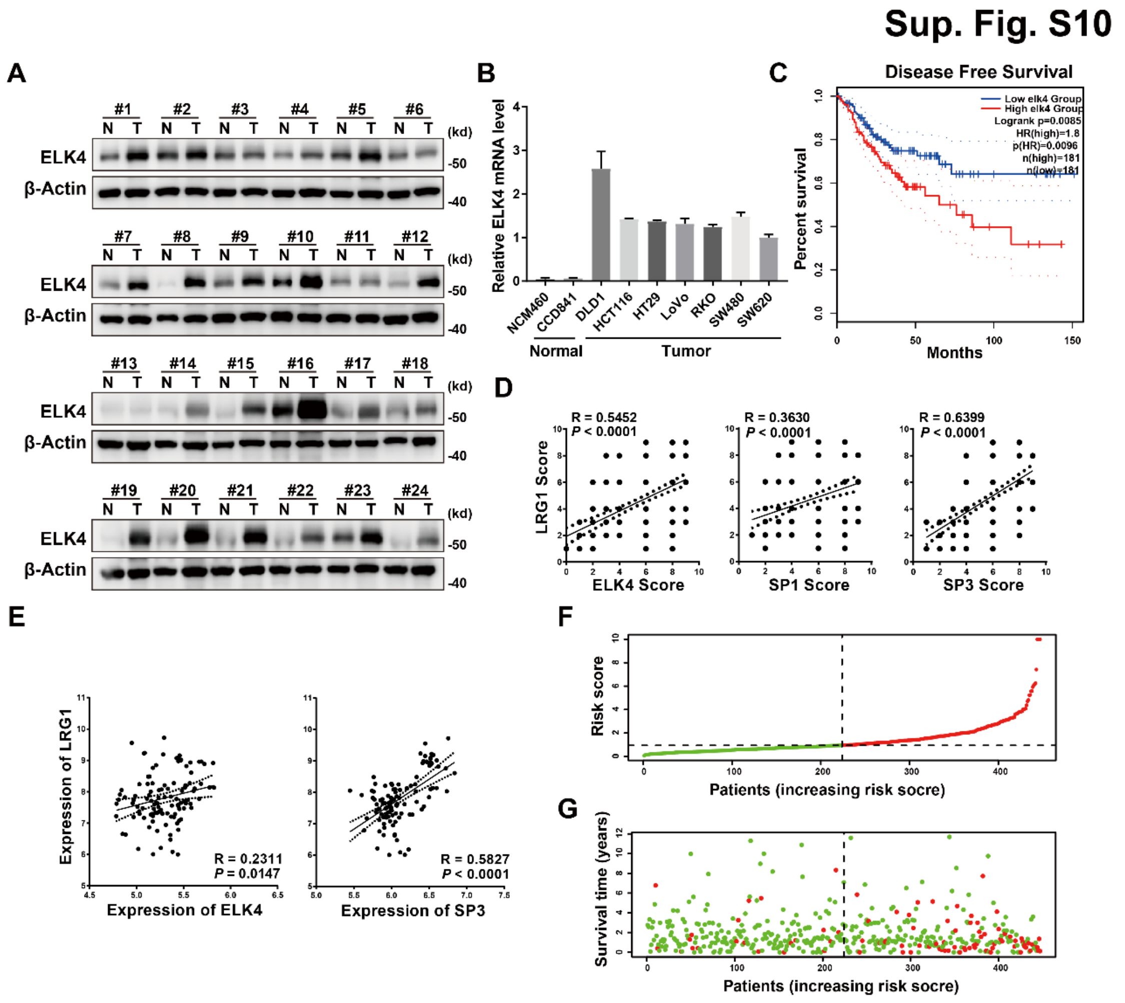

Supplementary Figure 10. Related to Figure 7.

(A) Western blot analysis of the protein level of ELK4 in 24 pairs of CRC and adjacent normal tissues.

(B) qPCR analysis of the mRNA levels of ELK4 in the two normal colonic epithelial cell lines and 7 CRC cell lines.

(C) Kaplan-Meier analysis of disease-free survival (DFS) for CRC patients in the TCGA CRC database stratified by ELK4 mRNA level.

(D) Positive correlations between LRG1 expression and ELK4, SP1 and SP3 expression in the CRC TMA cohort. Pearson correlation analysis was used to evaluate the associations.

(E) Correlations of the mRNA level of LRG1 with those of ELK4 and SP3 in GSE20916. Pearson correlation analysis was used to evaluate the association.

(F and G) The risk score distribution (F), and OS time and status (G) of patients in TCGA-COAD. The dotted line indicates the cutoff value for dividing the patients into

the low- and high-risk groups.

Supplementary Tables

Table S1. Differentially expressed genes (DEGs) in the ELK4 knockdown HCT116 cells.

gene_id	log2FoldChange	pval	padj	up_down
RASGRP3	-3.42469	2.41E-23	3.96E-19	Down
ELK4	-1.57613	8.48E-21	6.97E-17	Down
KRTAP3-1	-4.51675	7.55E-20	4.13E-16	Down
DYNLRB1	1.646171	1.14E-17	4.70E-14	Up
HOXA3	-2.20281	4.89E-17	1.61E-13	Down
CDKN1A	-1.26168	1.03E-15	2.83E-12	Down
ARL14EPL	1.563292	9.52E-15	1.96E-11	Up
SMAD7	-1.86096	8.49E-15	1.96E-11	Down
ZNF804A	-1.57096	3.23E-14	5.89E-11	Down
SULF2	-1.41489	1.83E-13	3.01E-10	Down
TMEM59L	-1.60746	8.31E-13	1.24E-09	Down
VGLL3	-2.629	9.18E-13	1.26E-09	Down
EVI5L	1.444411	2.00E-12	2.53E-09	Up
KRT34	-3.53014	7.09E-12	8.32E-09	Down
HSD17B10	1.22649	8.61E-12	8.84E-09	Up
ID3	1.220895	8.55E-12	8.84E-09	Up
LRG1	-2.6233	1.02E-11	9.80E-09	Down
RGS2	1.323999	1.07E-11	9.80E-09	Up
CCND1	-1.00282	1.82E-10	1.57E-07	Down
ANKRD46	-1.14478	6.10E-10	5.01E-07	Down
MAPRE3	1.531748	8.94E-10	6.68E-07	Up
C6orf141	1.628845	1.10E-09	7.85E-07	Up
APOBEC3B	-1.1833	2.74E-09	1.87E-06	Down
TMED4	1.017567	8.32E-09	5.26E-06	Up
CCDC85B	1.04453	9.37E-09	5.31E-06	Up
GALNT5	-1.72824	9.16E-09	5.31E-06	Down
KRTAP2-3	-1.9571	8.84E-09	5.31E-06	Down
ALPP	-2.707	1.28E-08	7.01E-06	Down
SUN2	1.01098	1.58E-08	8.39E-06	Up
OLFML3	-2.87932	2.46E-08	1.26E-05	Down
LAMA3	-1.13061	3.29E-08	1.64E-05	Down
AFAP1L1	-1.21481	4.65E-08	2.25E-05	Down
FGFBP1	1.304607	1.19E-07	5.27E-05	Up
MCAM	-1.22117	1.30E-07	5.63E-05	Down
SDCBP2	-1.31155	2.09E-07	8.79E-05	Down
ADGRF4	-2.20294	5.47E-07	0.000189	Down
TNFRSF10C	-1.1584	6.34E-07	0.000204	Down
ID1	1.162439	7.64E-07	0.000237	Up
ANKRD10	1.00211	1.65E-06	0.000475	Up

LONRF3	-1.02253	2.24E-06	0.000635	Down
ALDOC	1.221365	3.04E-06	0.000816	Up
SYNPO	1.021305	3.23E-06	0.000842	Up
RFTN1	-1.33171	3.30E-06	0.000848	Down
ST14	-1.12222	3.73E-06	0.000929	Down
KRT18	-1.03595	4.00E-06	0.00098	Down
RBP1	-1.5515	4.08E-06	0.000985	Down
PXDN	-1.23567	5.76E-06	0.001324	Down
SUSD1	-1.01027	6.30E-06	0.0014	Down
IFI6	-1.06817	7.98E-06	0.001682	Down
TACSTD2	-1.78447	8.46E-06	0.001757	Down
TNNC1	-1.43043	8.55E-06	0.001757	Down
FLNC	-1.04572	8.79E-06	0.001784	Down
SNX15	1.083715	1.04E-05	0.002052	Up
HIST2H2BE	-1.64606	1.66E-05	0.003036	Down
RAB26	1.245644	1.88E-05	0.003397	Up
MAN1A1	1.009898	2.45E-05	0.004377	Up
ANGPT2	2.267238	2.53E-05	0.004466	Up
ANK2	1.086327	3.05E-05	0.00533	Up
TLR3	-1.34672	3.51E-05	0.005954	Down
SYBU	1.010186	3.56E-05	0.005968	Up
HIST1H2BC	-1.80697	3.92E-05	0.006442	Down
SERPINB7	-1.75381	3.98E-05	0.006465	Down
ANKDD1A	1.094723	0.0001	0.014202	Up
KLHDC7A	-1.9624	0.000105	0.014585	Down
ALPK2	-2.60023	0.000108	0.014791	Down
LMO2	-1.40062	0.000108	0.014791	Down
ZNF488	1.066852	0.000132	0.017479	Up
CYP4F12	1.399796	0.000135	0.017614	Up
AGAP11	1.274552	0.000146	0.018173	Up
LRP1B	1.633264	0.00016	0.019483	Up
ETV1	1.603631	0.000191	0.021857	Up
YPEL1	1.975477	0.000214	0.023556	Up
DISC1	-1.18351	0.000234	0.024758	Down
JUND	1.117287	0.000301	0.029255	Up
SPC24	1.206279	0.000307	0.029666	Up
CORO1A	1.039932	0.000318	0.030172	Up
C2orf54	-3.1166	0.000328	0.030955	Down
GPRC5A	-1.08846	0.000347	0.03243	Down
GPR15	2.808217	0.000391	0.034391	Up
TP53I3	-1.10168	0.000408	0.035694	Down
ESR1	-4.36406	0.000427	0.036452	Down
CCDC80	-1.57913	0.000469	0.038138	Down
TENM4	-3.57719	0.000482	0.039043	Down

LBH	-1.71451	0.000528	0.041502	Down
THBS1	-1.12747	0.000533	0.041689	Down
RAB15	-1.14574	0.000619	0.046214	Down
TNXB	1.01109	0.000716	0.050709	Up
CAPN14	-5.12103	0.000833	0.055849	Down
SLC4A5	1.082252	0.000846	0.056534	Up
PRRT3	1.438296	0.000893	0.058012	Up
HOXA4	-1.72573	0.000925	0.059577	Down
SPATA18	-1.35198	0.00097	0.061761	Down
KRT17	-3.23019	0.000981	0.062118	Down
PLXNC1	-4.17779	0.0012	0.071989	Down
LBHD1	-1.11332	0.001429	0.081113	Down
WFDC3	-1.3891	0.001432	0.081113	Down
GSTA4	1.433037	0.001628	0.088602	Up
PNMA2	-1.4329	0.001623	0.088602	Down
LCN2	-1.44054	0.001811	0.093568	Down
KIAA1549L	-1.12545	0.00187	0.095414	Down
VAMP7	-1.97142	0.002063	0.10264	Down
SRPX2	-1.03767	0.002159	0.105565	Down
AKR1B10	-1.09197	0.002222	0.107734	Down
CALCRL	1.219429	0.002574	0.121884	Up
KCNH3	1.038102	0.002636	0.123274	Up
LRRN4	-1.5054	0.002641	0.123274	Down
H6PD	1.165171	0.002993	0.134374	Up
ASPRV1	1.553512	0.003302	0.144696	Up
KLRC2	1.501238	0.003588	0.152751	Up
FPR1	-2.87402	0.003665	0.155599	Down
SCN3B	-1.2748	0.003677	0.155713	Down
TNFSF18	Inf	0.00437	0.174848	Up
SHC2	-1.3325	0.004461	0.1762	Down
PPIL6	1.100848	0.004615	0.179707	Up
SSBP2	1.180792	0.004745	0.182681	Up
KRT20	-1.25787	0.004807	0.183286	Down
ATP6V0A4	-1.66294	0.004988	0.18929	Down
COX6B2	1.616384	0.005211	0.193728	Up
KRBA2	1.234654	0.005237	0.193833	Up
CYP2C8	-2.18161	0.005266	0.194465	Down
HTR6	1.241465	0.005402	0.197681	Up
LIPG	-1.12472	0.005454	0.198358	Down
DDIT4	1.516781	0.006264	0.215606	Up
NLRP5	-1.71658	0.006276	0.215606	Down
SGIP1	-1.53074	0.006383	0.218518	Down
MYO15B	1.618038	0.006721	0.224941	Up
ETV7	-1.47127	0.007221	0.236187	Down

PELI2	-1.01331	0.007583	0.244471	Down
RASA4	1.49198	0.008115	0.256064	Up
GAST	-1.41393	0.008405	0.26108	Down
GPSM3	-1.46711	0.008499	0.262019	Down
FAT2	-1.79123	0.00892	0.266319	Down
TNFRSF6B	1.062089	0.008995	0.266319	Up
ANKK1	2.062313	0.01019	0.280946	Up
NLRP4	-1.24421	0.010515	0.287017	Down
PTPRZ1	2.754125	0.011583	0.299455	Up
LRRIQ1	1.317618	0.011879	0.301685	Up
SNTB1	-1.03745	0.011871	0.301685	Down
TXNRD3NB	Inf	0.011764	0.301685	Up
HIST1H2BD	-1.00215	0.012474	0.310681	Down
FGF1	-1.49925	0.013714	0.327834	Down
SOCS1	1.203361	0.013704	0.327834	Up
DPP4	1.367077	0.014212	0.334562	Up
PHOSPHO2	1.145398	0.014159	0.334562	Up
DEPP1	-1.06312	0.01437	0.337807	Down
FAM57B	2.855878	0.014391	0.337819	Up
HLA-DRB1	-1.22203	0.015177	0.347931	Down
GSTA2	2.004166	0.015325	0.348777	Up
HIST1H3E	-1.54067	0.015511	0.350757	Down
MPP4	-1.9309	0.015913	0.355687	Down
CUBN	1.22625	0.016072	0.356891	Up
CREB3L3	1.357879	0.016205	0.358377	Up
ALPK3	1.493635	0.016666	0.362518	Up
BHMG1	-1.98638	0.016637	0.362518	Down
TMEM253	-2.30098	0.01706	0.36836	Down
DRICH1	1.077264	0.017254	0.369782	Up
COL6A3	-1.28551	0.017876	0.373209	Down
ANKRD37	2.205161	0.018463	0.380652	Up
TEX36	-1.8173	0.019271	0.389026	Down
CLEC4A	1.538967	0.019353	0.389111	Up
LRRC73	1.425358	0.020734	0.403125	Up
CYSRT1	1.209102	0.021792	0.411601	Up
MILR1	1.164788	0.021861	0.412427	Up
SELENOP	1.222567	0.022116	0.414291	Up
ATOH8	1.379468	0.022639	0.417991	Up
SAMD11	1.237702	0.023337	0.422007	Up
CARD9	-1.52262	0.023578	0.425275	Down
PPP1R1A	-1.92787	0.024312	0.430954	Down
DAPP1	-1.21758	0.024373	0.431575	Down
CALHM5	-1.30778	0.024664	0.434387	Down
S100A5	1.292767	0.024722	0.434599	Up

TRHDE	-1.48547	0.025248	0.438842	Down
ME3	-2.78788	0.025435	0.43949	Down
TMEM225B	1.210995	0.025676	0.443175	Up
SPATC1L	-1.73585	0.026022	0.446337	Down
IRS4	-3.42129	0.026605	0.451329	Down
GPR78	#NAME?	0.027442	0.461549	Down
SCEL	-1.76633	0.027781	0.465806	Down
SLCO2A1	-2.41475	0.027995	0.467012	Down
NT5M	1.001339	0.028335	0.470301	Up
MMP20	-1.81521	0.028702	0.473129	Down
KATNAL1	1.175218	0.029254	0.47642	Up
PPFIA4	1.531965	0.029457	0.477362	Up
ALPL	-1.17488	0.029947	0.477665	Down
ASB2	Inf	0.031457	0.487651	Up
FTCD	3.068696	0.032211	0.493143	Up
SPACA6	1.037059	0.033538	0.501	Up
TNFSF4	1.019836	0.034034	0.503867	Up
FAM228B	1.143716	0.034206	0.505011	Up
MTMR7	-2.97054	0.036182	0.517487	Down
LOC101928635	2.413605	0.036827	0.522521	Up
ERBB4	3.733676	0.037944	0.532449	Up
MYL7	#NAME?	0.040099	0.548637	Down
PHACTR3	#NAME?	0.040099	0.548637	Down
LOC101928722	1.34355	0.041278	0.556886	Up
CLCA2	#NAME?	0.041783	0.558852	Down
ANO7	1.390213	0.042477	0.561979	Up
IFNE	-1.26387	0.043045	0.56591	Down
COL5A3	1.439946	0.045146	0.574997	Up
FER1L5	1.103277	0.044831	0.574997	Up
ALDH1L2	1.005207	0.045237	0.575341	Up
GJA1	2.468826	0.045439	0.576459	Up
PLA2G2F	Inf	0.045669	0.578139	Up
LNP1	1.325519	0.048356	0.590127	Up
MMP24	-2.82608	0.048294	0.590127	Down
ASGR1	-1.47954	0.048835	0.592218	Down
TWIST1	1.879985	0.048911	0.592635	Up
MYO15A	1.124709	0.049305	0.594544	Up
ACTBL2	-1.84953	0.049432	0.595063	Down

Table S2. The gene list of each GO category.

Term description	Genes	p adjust
Signal transduction	KLRC2, ERBB4, SNX15, FPR1, GJA1, TLR3, GAST, TNFSF18, GPRC5A, TMED4, ANK2, DAPP1, PPP1R1A, FGF1, ANKDD1A, ANGPT2, RASA4, FGFBP1, IRS4, TRHDE, TNFSF4, ESR1, TENM4, TNFRSF10C, KRT17, ASB2	3.91E-04
Angiogenesis	HOXA3, SRPX2, ID1, CALCRL, FGF1, MCAM, ANGPT2	0.028789
Negative regulation of sequence-specific DNA binding transcription factor activity	TNFSF4, SMAD7, ID1, ESR1, ID3, TWIST1	3.89E-04
Positive regulation of angiogenesis	LRG1, THBS1, FGF1, ANGPT2, TWIST1	0.031716
Tumor necrosis factor-mediated signaling pathway	TNFRSF6B, TNFRSF10C, KRT18, TNFSF4, TNFSF18	0.034388
Response to exogenous dsRNA	CARD9, IFNE, TLR3, RFTN1	0.005169
Regulation of ventricular cardiac muscle cell membrane depolarization	SMAD7, SCN3B, GJA1	0.002167
Atrial cardiac muscle cell action potential	ANK2, SCN3B, GJA1	0.003664
Mammary gland alveolus development	CCND1, ERBB4, ESR1	0.013109
Innate immune response in mucosa	HIST1H2BC, HIST1H2BD, HIST2H2BE	0.027397

Table S3. List of primary antibodies used in this study, and information on working dilutions of antibodies in Western blotting (WB), immunohistochemistry (IHC) and immunofluorescence (IF).

Antibody	Species	Source	Catalog#	Applications	Dilution
ELK4	Rabbit	Atlas Antibodies	HPA028863	WB<br>IHC	1:500<br>1:100
ELK4	Mouse	Abnova	H00002005-B01P	IF	1:100
SP1	Rabbit	ABclonal	A19649	WB<br>IHC<br>IF	1:1000<br>1:100<br>1:100
SP3	Rabbit	Abcam	ab227856	WB<br>IHC<br>IF	1:1000<br>1:100<br>1:100
LRG1	Rabbit	Atlas Antibodies	HPA001888	WB<br>IHC	1:1000<br>1:100
FLAG	Rabbit	Cell Signaling Technology	#14793	WB	1:1000
HA	Rabbit	Cell Signaling Technology	#3724	WB	1:1000
ERK	Rabbit	Cell Signaling Technology	#4695	WB	1:1000
p-ERK	Rabbit	Cell Signaling Technology	#4370	WB	1:1000
β-Actin	Rabbit	Abcam	ab8226	WB	1:10000
Ki67	Rabbit	Abcam	ab15580	IHC	1:100
Cleaved PARP	Rabbit	Cell Signaling Technology	#5625	WB<br>IHC	1:1000<br>1:100
CD31	Rabbit	Cell Signaling Technology	#77699	IHC	1:100
CD31	Rabbit	Abcam	ab28364	IHC	1:100
HIF-1α	Rabbit	Cell Signaling Technology	#48085	IHC	1:100
CD105	Rabbit	Abcam	ab221675	IHC	1:100

Table S4. Sequence of shRNA and siRNA used in this study.

shRNA/siRNA	Sequence (5’-3’)
shELK4_1	CCGGGACATTCAACATGATTGCATTCTCGAGAATGCAATCATGTTGAATGTCTTTTTG
shELK4_2	CCGGACACCATCTGTCATCAAATTTCTCGAGAAATTTGATGACAGATGGTGTTTTTTTG
shELK4_3	CCGGCAATGCTGATAATAGACTATTCTCGAGAATAGTCTATTATCAGCATTGTTTTTTG
siSP1	GAAGGGAGGCCCAGGUGUA
siSP3	GGUAUUCACUCUAGCAGUA

Table S5. Sequence of real-time PCR primers used in this study.

Gene	Forward Sequence 5' to 3'	Reverse Sequence 5' to 3'
ELK4	AGTGGGCAGGATTGAGGGT	GCCAGTTTCTCGGCTGGATT
SP1	GTGGAGGCAACATCATTGCTG	GCCACTGGTACATTGGTCACAT
SP3	AGTGGGCAGTATGTTCTTCCC	GACTGGATCTGTGGTATCACTTG
LRG1	GTTGGAGACCTTGCCACCT	GCTTGTTGCCGTTCAGGA
STC2	GCGTGCAGGTTCAGTGTGA	GGCCAGTCTCCCTACTGCT
LBHD1	ATTGGCAAGGTTGAAGGTCAC	GATTCCACCACAATAGACGGC
HOXA3	ATGCAAAAAGCGACCTACTACG	TACGGCTGCTGATTGGCATTA
SRPX2	TAGTGGCACTTACACCTGCAC	ATTCGGCTGCGATCACCTTC
MCAM	GGTCGCTACCTGTGTAGGGA	TGGACCCGGTTCTTCTCCT
β-ACTIN	GACCTGTACGCCAACACAG	CTCAGGAGGAGCAATGATC
Elk4	CGAGCCCTGCGATACTACTAC	CGTCAGTGGGTCCATTTTCAA
Lrg1	CCATGTCAGTGTGCAGATTC	AAGAGTGAGAGGTGGAAGAG
Gapdh	ACTGAGGACCAGGTTGTCTCC	CTGTAGCCGTATTCATTGTCATACC
LRG1-promoter1 (ChIP)	AGGTGCCGGTTAGGAGTTTGAA	CACCCTGGGGCCGTATTT
LRG1-promoter2 (ChIP)	CGCTTCATCCTCCCTCCTG	CTGAGGCTGGGTCCAAGG
LRG1-enhancer1 (ChIP)	TTGAATCTAGCGCTCCCGAA	CCGGAACTGACCAATGGGGA
LRG1-enhancer2 (ChIP)	TGAGCTGTGAGTGGACTGGG	GATTACAGGCATGAGACACTG
